# Intermedin Alleviates Diabetic Cardiomyopathy by Up-Regulating CPT-1β through Activation of the Phosphatidyl Inositol 3 Kinase/Protein Kinase B Signaling Pathway

**DOI:** 10.3390/ph17091204

**Published:** 2024-09-12

**Authors:** Jie Zhao, Ling Han, Ya-Rong Zhang, Shi-Meng Liu, Deng-Ren Ji, Rui Wang, Yan-Rong Yu, Mo-Zhi Jia, San-Bao Chai, Hui-Fang Tang, Wei Huang, Yong-Fen Qi

**Affiliations:** 1 Laboratory of Cardiovascular Bioactive Molecule, School of Basic Medical Sciences, Peking University, Beijing 100083, China1510305113@pku.edu.cn (D.-R.J.); wang-rui@bjmu.edu.cn (R.W.); yuyr@bjmu.edu.cn (Y.-R.Y.); mzhjia@bjmu.edu.cn (M.-Z.J.); 2 State Key Laboratory of Vascular Homeostasis and Remodeling, Peking University, Beijing 100083, China; 3 Department of Pathogen Biology, School of Basic Medical Sciences, Peking University Health Science Center, Beijing 100083, China; 4 Department of Cardiology, Fuxing Hospital, Capital Medical University, Beijing 100038, China; hling966@sina.com; 5 Department of Endocrinology and Metabolism, Peking University International Hospital, Beijing 102206, China; chaisb@126.com; 6 Department of Cardiology Laboratory, First Affiliated Hospital of University of South China, Hengyang 421001, China; tanghuifang999@163.com; 7 Institute of Cardiovascular Sciences, State Key Laboratory of Vascular Homeostasis and Remodeling, School of Basic Medical Sciences, Peking University Health Science Center, Beijing 100083, China

**Keywords:** diabetic cardiomyopathy, intermedin, CPT-1β, PI3K/Akt

## Abstract

Diabetic cardiomyopathy (DCM), one of the most serious long-term consequences of diabetes, is closely associated with myocardial fatty acid metabolism. Carnitine palmitoyltransferase-1β (CPT-1β) is the rate-limiting enzyme responsible for β-oxidation of long-chain fatty acids. Intermedin (IMD) is a pivotal bioactive small molecule peptide, participating in the protection of various cardiovascular diseases. However, the role and underlying mechanisms of IMD in DCM are still unclear. In this study, we investigated whether IMD alleviates DCM via regulating CPT-1β. A rat DCM model was established by having rats to drink fructose water for 12 weeks. A mouse DCM model was induced by feeding mice a high-fat diet for 16 weeks. We showed that *IMD* and its receptor complexes levels were significantly down-regulated in the cardiac tissues of DCM rats and mice. Reduced expression of *IMD* was also observed in neonatal rat cardiomyocytes treated with palmitic acid (PA, 300 μM) in vitro. Exogenous and endogenous IMD mitigated cardiac hypertrophy, fibrosis, dysfunction, and lipid accumulation in DCM rats and IMD-transgenic DCM mice, whereas knockout of IMD worsened these pathological processes in IMD-knockout DCM mice. In vitro, IMD alleviated PA-induced cardiomyocyte hypertrophy and cardiac fibroblast activation. We found that CPT-1β enzyme activity, mRNA and protein levels, and acetyl-CoA content were increased in T2DM patients, rats and mice. IMD up-regulated the CPT-1β levels and acetyl-CoA content in T2DM rats and mice. Knockdown of CPT-1β blocked the effects of IMD on increasing acetyl-CoA content and on inhibiting cardiomyocyte hypertrophy and cardiac fibroblast activation. IMD receptor antagonist IMD_17–47_ and the phosphatidyl inositol 3 kinase (PI3K)/protein kinase B (Akt) inhibitor LY294002 reversed the effects of IMD on up-regulating CPT-1β and acetyl-CoA expression and on inhibiting cardiomyocyte hypertrophy and cardiac fibroblast activation. We revealed that IMD alleviates DCM by up-regulating CPT-1β via calcitonin receptor-like receptor/receptor activity-modifying protein (CRLR/RAMP) receptor complexes and PI3K/Akt signaling. IMD may serve as a potent therapeutic target for the treatment of DCM.

## 1. Introduction

Diabetes has become one of the most prevalent and serious chronic diseases, and it is a metabolic disorder characterized by hyperglycemia, resulting from impaired insulin secretion and/or action [1,2]. One of the most common complications of diabetes mellitus is diabetic cardiomyopathy (DCM), which is the leading cause of death in diabetic patients [3,4,5,6]. DCM refers to cardiac structural and functional abnormalities caused by disturbance in myocardial glucose and lipid metabolism in diabetic patients, regardless of other factors such as coronary artery disease, valvular disease, and hypertension [2,4]. DCM is characterized by cardiac hypertrophy, cardiac fibrosis, several perturbations in myocardial energy metabolism, and left ventricular (LV) diastolic dysfunction with normal ejection fraction (EF) evolving to LV systolic dysfunction with reduced EF [6,7,8,9]. Increased fatty acid uptake surpasses fatty acid oxidation, resulting in a large amount of lipid accumulation and overload in the diabetic heart. These pathophysiological abnormalities cause LV diastolic and systolic dysfunction, promoting the occurrence and development of DCM [10,11,12]. Increasing myocardial fatty acid oxidation may offer a potential strategy to prevent DCM or heart failure via reducing lipid accumulation [11,12,13]. Currently, there are no effective therapeutic targets to protect against DCM. Therefore, it is urgent to discover promising therapeutic targets for DCM.

Carnitine palmitoyltransferase-1 (CPT-1) is the rate-limiting enzyme responsible for β-oxidation of long-chain fatty acids. CPT-1 has two isoforms, CPT-1α and CPT-1β, that are located in the outer mitochondrial membrane. CPT-1β is the primary CPT-1 isoform in the heart. CPT-1β mediates the transport of fatty acyl CoA into the mitochondria for myocardial fatty acid β-oxidation and then generates acetyl-CoA [14,15,16,17,18]. A previous report demonstrated that CPT-1β expression was elevated in the heart of T2DM mice [17]. Overexpression of CPT-1 can enhance fatty acid oxidation, reduce lipid accumulation, and improve insulin sensitivity in high-fat-diet (HFD)-fed rats [18]. Deletion or inhibition of CPT-1β can lead to myocardial lipid accumulation, reduced insulin sensitivity, cardiac hypertrophy, cardiac fibrosis, and cardiac dysfunction in mice [19,20,21,22,23]. These findings suggest that up-regulating CPT-1β might be a possible therapeutic target for alleviating DCM.

Many studies have demonstrated that multiple bioactive molecules can regulate CPT-1β expression, such as leptin [24], adiponectin [25], and eicosapentaenoic acid [26] up-regulating the mRNA levels of *Cpt1b* in the hearts of rats, C_2_C_12_ myocytes, and primary human adipose-tissue-derived stem cells, respectively. By contrast, angiotensin II [27] down-regulates the protein levels of CPT-1 in adult rat cardiomyocytes. Intermedin (IMD), also known as adrenomedullin 2 (ADM2), is a member of the calcitonin gene-related peptide (CGRP) superfamily discovered in 2004, which is a paracrine/autocrine bio-active polypeptide [28,29]. IMD_1–53_ is the longest active fragment, which is produced by proteolytic cleavage of human prepro-IMD [30,31,32,33,34]. IMD exists in various cardiovascular cells, including cardiomyocytes, fibroblasts, vascular smooth muscle cells, and endothelial cells [30,31,32,33,34]. IMD binds non-selectively to its receptor complexes, calcitonin receptor-like receptor/receptor activity-modifying proteins (CRLR/RAMPs), and activates post-receptor signaling pathways to exert biological effects [30,31,32,33,34]. IMD has a protective role in various cardiovascular diseases, such as cardiac hypertrophy, cardiac fibrosis, congestive heart failure, hypertension, atherosclerosis, and vascular calcification [30,34,35,36,37,38]. Moreover, IMD can reduce the levels of fasting blood glucose (FBG), insulin, free fatty acid, triglycerides, and total cholesterol, thereby improving metabolic syndromes like obesity and insulin resistance in T1DM rats, T2DM mice, hyperhomocysteinemia mice, and ApoE^−/−^ atherosclerosis mice [39,40,41,42]. These results indicate that IMD may serve as a potent therapeutic target for the treatment of DCM. However, the role and possible mechanism of IMD in DCM remain unclear. 

In this study, we investigated the role and mechanism of IMD in DCM. We found that IMD expression is down-regulated in heart tissues of DCM rats and mice. Exogenous and endogenous IMD mitigated DCM, whereas knockout of IMD worsened DCM. Mechanistically, this study identified that IMD alleviates DCM by up-regulating CPT-1β via CRLR/RAMP receptor complexes and phosphatidyl inositol 3 kinase (PI3K)/protein kinase B (Akt) signaling, suggesting that targeting IMD is a potential therapeutic strategy for DCM.

## 2. Results

### 2.1. IMD Levels Are Significantly Down-Regulated in Cardiac Tissues of DCM Rats, DCM Mice, and Hypertrophic Cardiomyocytes

We established a rat DCM model by having rats to drink fructose drinking water for 12 weeks. To reveal the potential role of exogenous IMD in DCM, we first examined the expression of IMD and its receptor complexes in the cardiac tissues of fructose-drinking rats. Cardiac mRNA levels of *IMD* and mRNA and the protein levels of its receptor complexes, CRLR/RAMPs, were decreased in fructose-drinking rats (Figure S1A,B). In vitro, we observed palmitic acid (PA)-induced cardiomyocyte hypertrophy (Figure S1C) and cardiac fibroblast activation of rats (Figure S1D). The *IMD* mRNA levels were decreased in PA-treated neonatal rat cardiomyocytes (NRCMs) (Figure S1E). These findings indicated that IMD is involved in DCM in fructose-drinking rats through its receptor complexes. We also examined the expression of IMD and its receptor complexes in the cardiac tissues of HFD-fed wildtype (WT) mice. Cardiac mRNA levels of *IMD* and mRNA and the protein levels of its receptor complexes, CRLR/RAMPs, were decreased in HFD-fed WT mice (Figure S2A,B), indicating that IMD is involved in DCM in HFD-fed mice through its receptor complexes. 

### 2.2. Exogenous IMD Alleviates DCM in Rats

**Figure 1:**
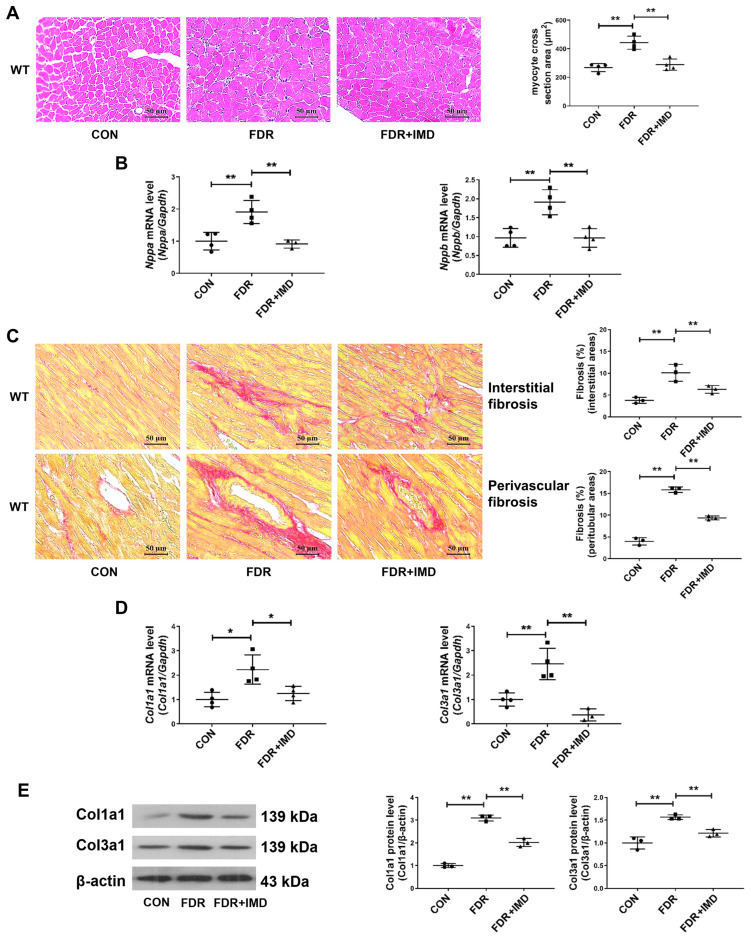

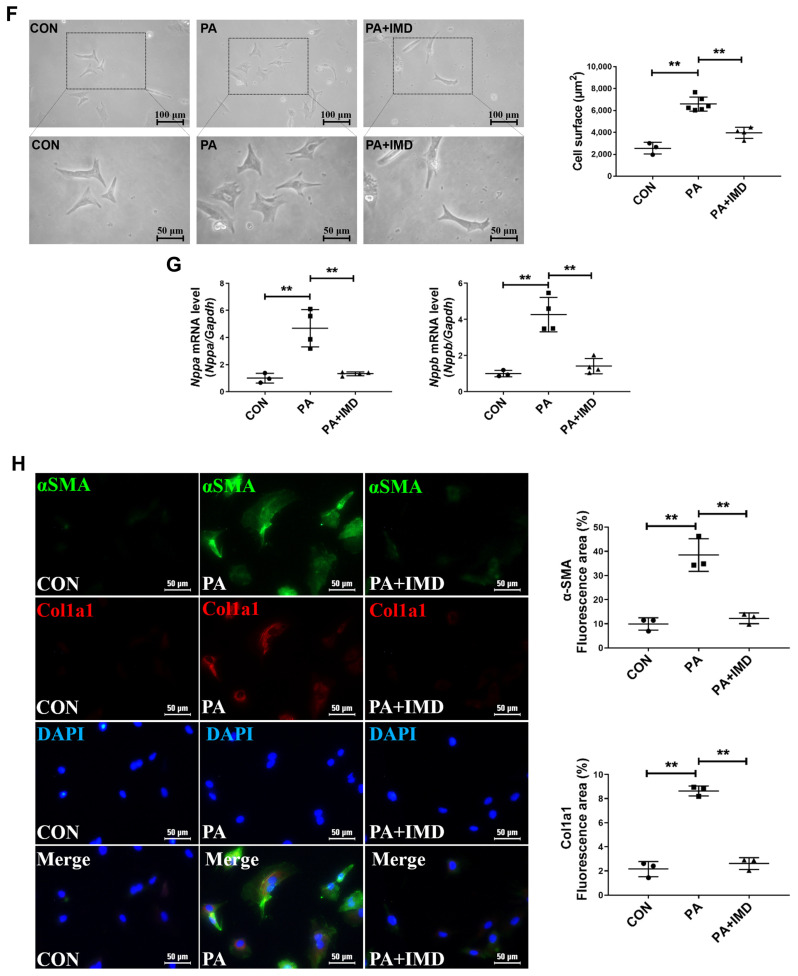
Exogenous IMD alleviates DCM in rats. (**A**) Hematoxylin–eosin staining of representative heart sections and cardiomyocyte cross-sectional area quantification in rats. Scale bar: 50 μm. *n* = 4. (**B**) Quantitative real-time PCR analysis of *Nppa* and *Nppb* mRNA expression in the hearts of diabetic rats. *n* = 3–4. (**C**) Sirius red staining of myocardial interstitial and perivascular fibrosis area with representative images and quantification in rats. Scale bar: 50 μm. *n* = 3. (**D**) Quantitative real-time PCR analysis of *Col1a1* and *Col3a1* mRNA expression in DCM rat hearts. *n* = 3–4. (**E**) Western blot analysis of Col1a1 and Col3a1 protein levels in DCM rat hearts. *n* = 3. (**F**) Representative images and quantification of surface (μm^2^) in NRCMs analyzed by ImageJ 1.53k. Scale bar: 100 μm, 50 μm. *n* = 3–6. (**G**) Quantitative real-time PCR analysis of *Nppa* and *Nppb* mRNA expression in NRCMs. *n* = 3–4. (**H**) Representative images and quantification of immunofluorescence staining for αSMA (green), Col1a1 (red) and DAPI (blue) in primary cultured rat cardiac fibroblasts. Merged images are shown. Scale bar: 50 μm. *n* = 3. Data are mean ± SD, * *p* < 0.05, ** *p* < 0.01.

Firstly, we constructed the DCM model by using fructose-drinking rats and assessed the influence of exogenous IMD on rat DCM in different dimensions. IMD treatment significantly reduced fructose-drinking-induced cardiac hypertrophy in rats, as shown by a reduced myocardial cross-sectional area and down-regulated mRNA levels of pro-hypertrophic genes natriuretic peptide A (*Nppa*) and natriuretic peptide B (*Nppb*) (Figure 1A,B). IMD administration also attenuated cardiac fibrosis induced by fructose-drinking in rats, as shown by a decreased cardiac interstitial and perivascular fibrosis area and down-regulated mRNA and protein levels of pro-fibrotic gene collagen type I alpha 1 chain (Col1a1) and collagen type III alpha 1 chain (Col3a1) (Figure 1C–E). Moreover, IMD administration significantly decreased FBG, fasting serum insulin levels, insulin resistance, serum triglycerides, and LDL-C levels, and it increased the serum HDL-C levels of rats (Table S1). These findings demonstrated that exogenous IMD mitigates DCM induced by fructose drinking.

In vitro, we investigated how IMD influenced PA-induced cardiomyocyte hypertrophy and cardiac fibroblast activation in rats. It was shown that IMD treatment reduced cell surface area and *Nppa* and *Nppb* mRNA levels of NRCMs treated with PA (Figure 1F,G). IMD treatment also down-regulated α-SMA and Col1a1 levels of rat cardiac fibroblast treated with PA (Figure 1H). These results suggested that IMD administration alleviated PA-induced cardiomyocyte hypertrophy and cardiac fibroblast activation in rats. 

### 2.3. IMD Overexpression Alleviates DCM in Mice 

**Figure 2:**
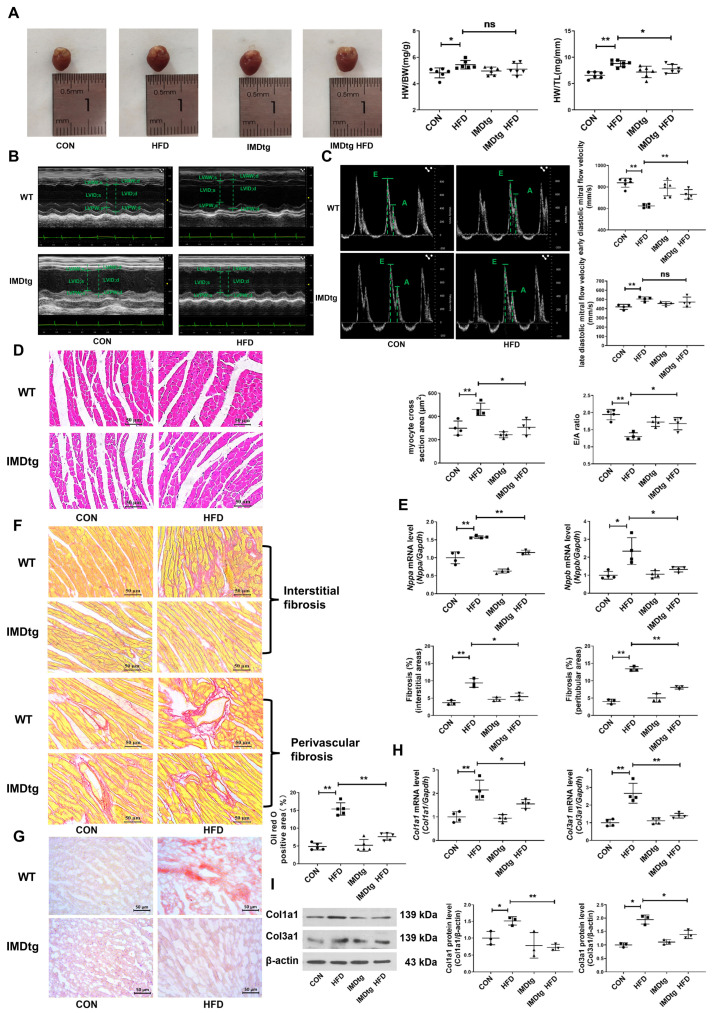
IMD overexpression alleviates DCM in mice. (**A**) Representative images of hearts, HW/BW ratio, and HW/TL ratio of diabetic WT and IMDtg mice. Scale bar: 10 mm. *n* = 6–7. (**B**) Representative echocardiographic images of diabetic WT and IMDtg mice. (**C**) Representative pulsed Doppler echocardiography pictures, early diastolic mitral flow velocity (**E**), late diastolic mitral flow velocity (**A**), and relative quantification of the mitral E/A ratio of diabetic WT and IMDtg mice. *n* = 4–6. (**D**) Hematoxylin–eosin staining of representative heart sections and cardiomyocyte cross-sectional area quantification in mice. Scale bar: 50 μm. *n* = 4. (**E**) Quantitative real-time PCR analysis of *Nppa* and *Nppb* mRNA expression in the hearts of diabetic WT and IMDtg mice. *n* = 3–4. (**F**) Sirius red staining of myocardial interstitial and perivascular fibrosis area with representative images and quantification from different mice. Scale bar: 50 μm. *n* = 3. (**G**) Oil Red O staining of myocardial interstitium with representative images and quantification from different mice. Scale bar: 50 μm. *n* = 5. (**H**) Quantitative real-time PCR analysis of *Col1a1* and *Col3a1* mRNA expression in the hearts of diabetic WT and IMDtg mice. *n* = 4. (**I**) Western blot analysis of Col1a1 and Col3a1 protein levels in the hearts of diabetic WT and IMDtg mice. *n* = 3. Data are mean ± SD, * *p* < 0.05, ** *p* < 0.01. ns: no significant difference.

Given the findings that exogenous IMD alleviates DCM, we next explored whether endogenous IMD has beneficial effects on DCM of IMD-transgenic (IMDtg) mice induced by an HFD. Firstly, quantitative real-time PCR results indicated that IMD was significantly overexpressed in IMDtg mice hearts (Figure S3A). Next, we assessed cardiac function and cardiac pathological morphology in each group of mice. IMD overexpression reduced the heart weight/tibial length (HW/TL) ratio, whereas the heart weight/body weight (HW/BW) ratio had no statistically significant changes (Figure 2A). Echocardiography and Doppler echocardiography showed that IMD overexpression decreased the LV wall thickness and increased LV EF, fractional shortening (FS), and the E/A ratio in IMDtg mice. These results suggested that IMD overexpression mitigated induced cardiac systolic and diastolic dysfunction in HFD-fed mice (Figure 2B,C and Table S2). Meanwhile, we found that IMD overexpression also attenuated cardiac hypertrophy, fibrosis, and lipid accumulation in IMDtg mice myocardium, as evidenced by the reduction in the myocardial cross-sectional area, pro-hypertrophic gene expression, interstitial and perivascular fibrosis area, Oil Red O positive area, and pro-fibrotic gene expression (Figure 2D–I). The prevalence of hypertension in T2DM individuals was up to three times higher than in non-diabetic individuals [43,44]. IMD also improved hypertension in HFD-fed IMDtg mice (Table S3). Furthermore, IMD overexpression significantly decreased fasting serum insulin levels, insulin resistance, serum levels of triglycerides, total cholesterol, LDL-C, and increased serum HDL-C levels in HFD-fed IMDtg mice versus HFD-fed WT mice (Table S4). These data indicated that endogenous IMD mitigated DCM induced by an HFD. 

### 2.4. IMD Deficiency Exacerbates DCM in Mice

**Figure 3:**
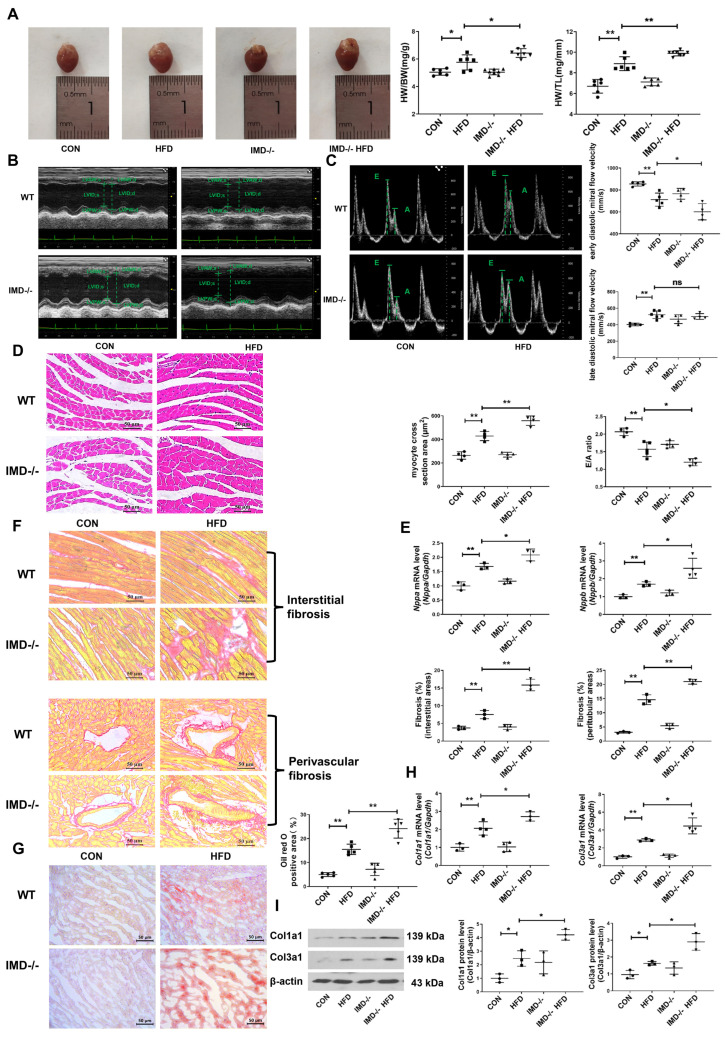
IMD deficiency exacerbates DCM in mice. (**A**) Representative images of hearts, the HW/BW ratio, and HW/TL ratio of diabetic WT and IMD^−/−^ mice. Scale bar: 10 mm. *n* = 6–8. (**B**) Representative echocardiographic images of diabetic WT and IMD^−/−^ mice. (**C**) Representative pulsed Doppler echocardiography pictures, early diastolic mitral flow velocity (**E**), late diastolic mitral flow velocity (**A**), and relative quantification of the mitral E/A ratio of diabetic WT and IMD^−/−^ mice. *n* = 4–5. (**D**) Hematoxylin–eosin staining of representative heart sections and cardiomyocyte cross-sectional area quantification in mice. Scale bar: 50 μm. *n* = 4. (**E**) Quantitative real-time PCR analysis of *Nppa* and *Nppb* mRNA expression in the hearts of diabetic WT and IMD^−/−^ mice. *n* = 3–4. (**F**) Sirius red staining of myocardial interstitial and perivascular fibrosis area with representative images and quantification in mice. Scale bar: 50 μm. *n* = 3. (**G**) Oil red O staining of myocardial interstitium with representative images and quantification from different mice. Scale bar: 50 μm. *n* = 5. (**H**) Quantitative real-time PCR analysis of *Col1a1* and *Col3a1* mRNA expression in the hearts of diabetic WT and IMD^−/−^ mice. *n* = 3–4. (**I**) Western blot analysis of Col1a1 and Col3a1 protein levels in the hearts of diabetic WT and IMD^−/−^ mice. *n* = 3. Data are mean ± SD, * *p* < 0.05, ** *p* < 0.01. ns: no significant difference.

To further confirm the cardiac protective effects of endogenous IMD on DCM, HFD-fed, IMD-knockout (IMD^−/−^) mice were used. Firstly, quantitative real-time PCR results indicated that IMD was strongly down-regulated in IMD^−/−^ mice hearts (Figure S3B). Then, we assessed cardiac function and cardiac pathological morphology in each group of mice. IMD deficiency increased the HW/BW ratio and HW/TL ratio in HFD-fed, IMD^−/−^ mice versus HFD-fed WT mice (Figure 3A). Echocardiography revealed that IMD deficiency significantly exacerbated cardiac systolic and diastolic dysfunction in HFD-fed, IMD^−/−^ mice (Figure 3B,C and Table S5). Consistent with this, the results of hematoxylin and eosin (H&E) staining, quantitative real-time PCR, and Sirius red and Oil Red O staining confirmed that IMD deficiency effectively aggravated cardiac hypertrophy, fibrosis, and lipid accumulation in the myocardium of HFD-fed, IMD^−/−^ mice (Figure 3D–I). IMD deficiency further exacerbated the hypertension of HFD-fed, IMD^−/−^ mice (Table S6). Moreover, IMD deficiency increased FBG, fasting serum insulin levels, insulin resistance, serum levels of triglycerides, and total cholesterol in HFD-fed, IMD^−/−^ mice versus HFD-fed WT mice (Table S7). Taken together, these results suggested that endogenous IMD deficiency aggravated the HFD-induced DCM of IMD^−/−^ mice. 

### 2.5. IMD Alleviates DCM by Up-Regulating CPT-1β 

**Figure 4:**
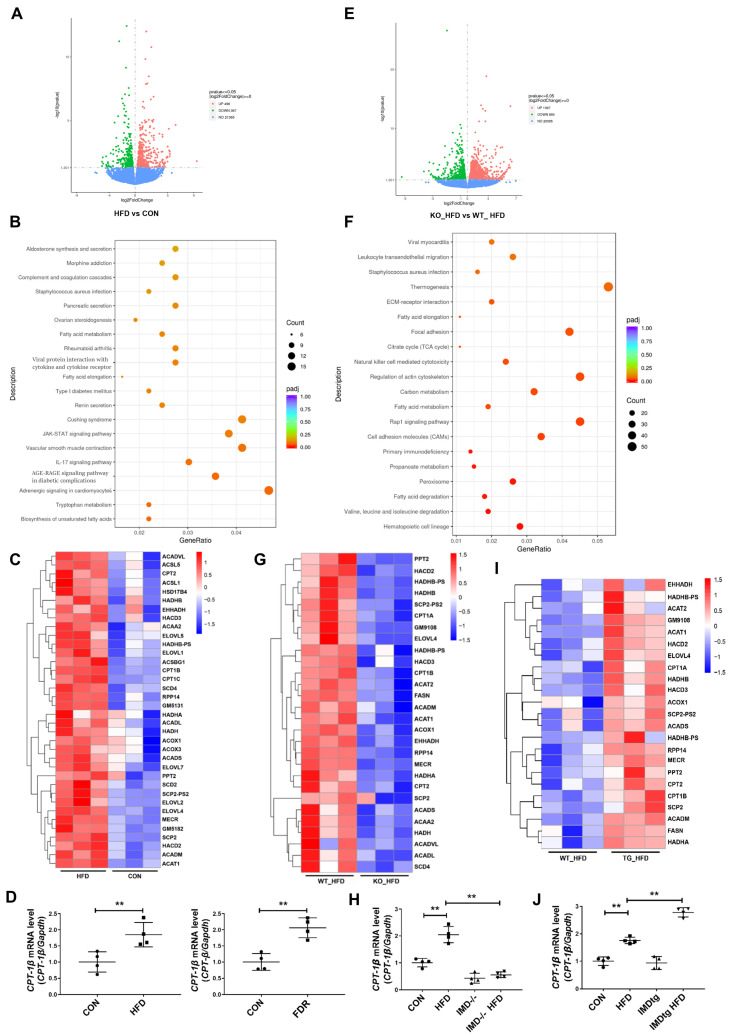
IMD up-regulates CPT-1β. RNA-sequencing analysis of hearts in mice. (**A**) Volcano plot showing the transcript expression profiles of differentially expressed genes in the hearts of the WT diabetic mice, respectively. *n* = 3. (**B**) KEGG pathway enrichment analysis showing the 20 most significantly enriched signaling pathways for differentially expressed genes in the hearts of WT diabetic mice. *n* = 3. (**C**) Heat map of the microarray results showing the 36 (ranked by *p*-values) differentially expressed fatty acid metabolism genes in hearts from the WT diabetic mice. Red, up-regulated; blue, down-regulated; white, no change. *n* = 3. (**D**) Quantitative real-time PCR analysis of *Cpt1b* mRNA expression in the hearts of WT diabetic mice and diabetic rats. *n* = 4. (**E**) Volcano plot showing the transcript expression profiles of differentially expressed genes in the hearts from the diabetic WT and IMD^−/−^ mice, respectively. *n* = 3. (**F**) KEGG pathway enrichment analysis showing the 20 most significantly enriched signaling pathways for differentially expressed genes in the hearts of diabetic WT and IMD^−/−^ mice. *n* = 3. (**G**) Heat map of the microarray results showing the 28 (ranked by *p*-values) differentially expressed fatty acid metabolism genes in hearts from the diabetic WT and IMD^−/−^ mice. Red, up-regulated; blue, down-regulated; white, no change. *n* = 3. (**H**) Quantitative real-time PCR analysis of *Cpt1b* mRNA expression in the hearts of diabetic WT and IMD^−/−^ mice. *n* = 4. (**I**) Heat map of the microarray results showing the 23 (ranked by P-values) differentially expressed fatty acid metabolism genes in hearts from the diabetic WT and IMDtg mice. Red, up-regulated; blue, down-regulated; white, no change. *n* = 3. (**J**) Quantitative real-time PCR analysis of *Cpt1b* mRNA expression in the hearts of diabetic WT and IMDtg mice. *n* = 4. Data are mean ± SD, ** *p* < 0.01.

**Figure 5:**
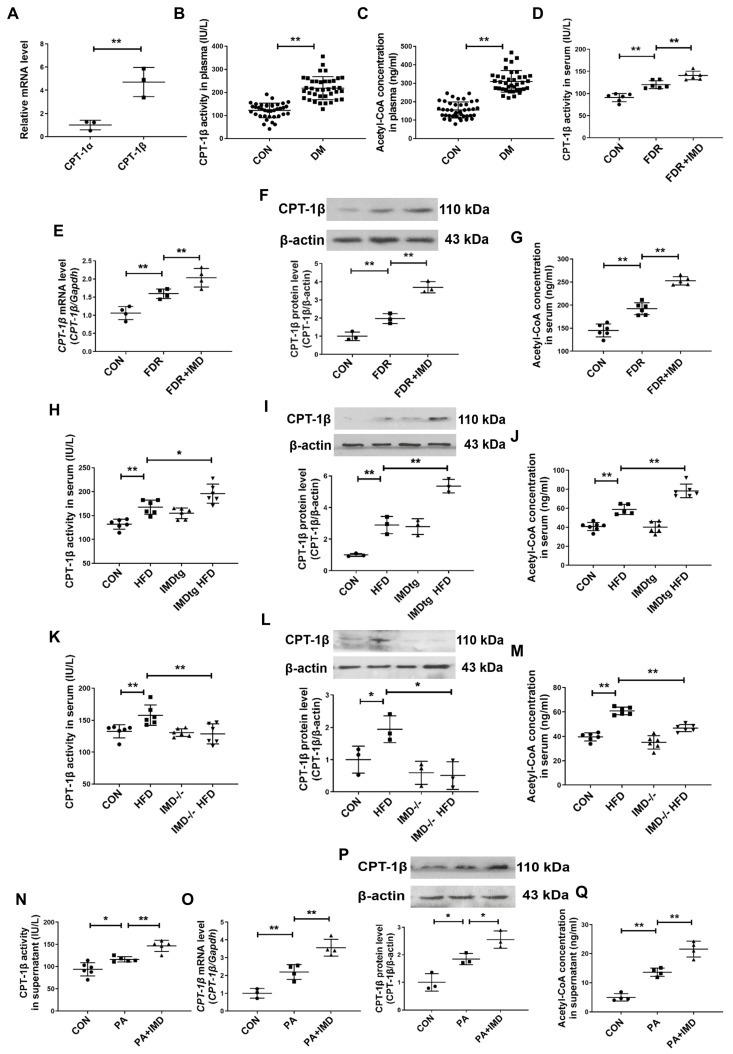

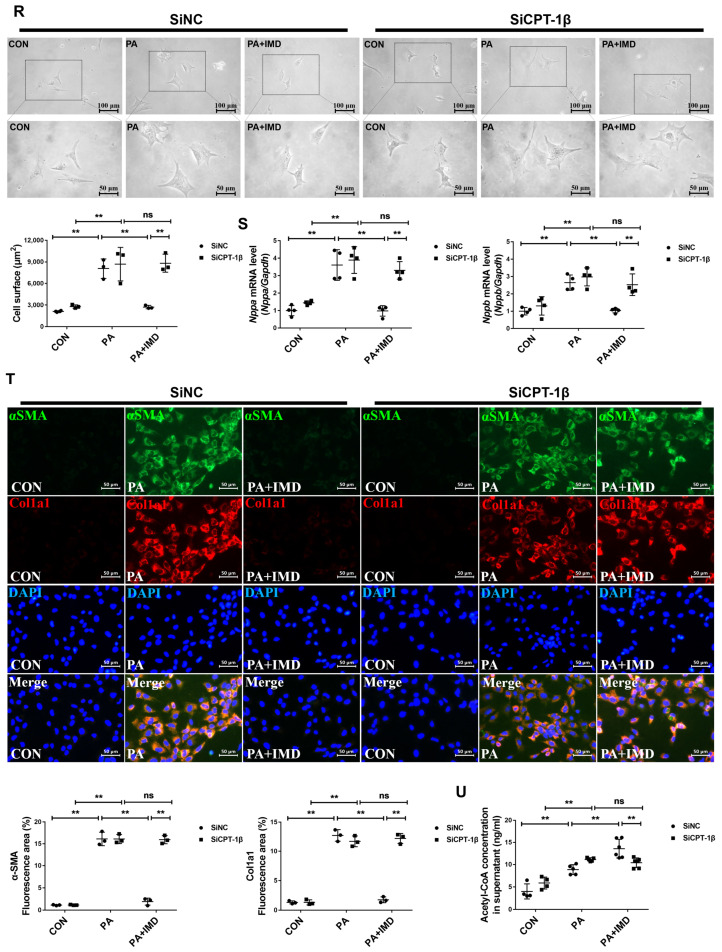
IMD alleviates DCM by up-regulating CPT-1β. (**A**) Quantitative real-time PCR analysis of *Cpt1a* and *Cpt1b* mRNA expression in NRCMs. *n* =3. (**B**) Enzyme-linked immunosorbent assay of plasma CPT-1β activity in T2DM patients and healthy controls. *n* = 38. (**C**) Enzyme-linked immunosorbent assay of plasma acetyl-CoA concentration in T2DM patients and healthy controls. *n* = 38–41. (**D**) Enzyme-linked immunosorbent assay of serum CPT-1β activity in diabetic rats. *n* = 6. (**E**) Quantitative real-time PCR analysis of *Cpt1b* mRNA expression in the hearts of diabetic rats. *n* = 4. (**F**) Western blot analysis of CPT-1β protein levels in diabetic rat hearts. *n* = 3. (**G**) Enzyme-linked immunosorbent assay of serum acetyl-CoA concentration in diabetic rats. *n* = 5–6. (**H**) Enzyme-linked immunosorbent assay of serum CPT-1β activity of diabetic WT and IMDtg mice. *n* = 6. (**I**) Western blot analysis of CPT-1β protein levels in the hearts of diabetic WT and IMDtg mice. *n* = 3. (**J**) Enzyme-linked immunosorbent assay of serum acetyl-CoA concentration in diabetic WT and IMDtg mice. *n* = 5–6. (**K**) Enzyme-linked immunosorbent assay of serum CPT-1β activity of diabetic WT and IMD^−/−^ mice. *n* = 6. (**L**) Western blot analysis of CPT-1β protein levels in the hearts of diabetic WT and IMD^−/−^ mice. *n* = 3. (**M**) Enzyme-linked immunosorbent assay of serum acetyl-CoA concentration in diabetic WT and IMD^−/−^ mice. *n* = 6. (**N**) Enzyme-linked immunosorbent assay of NRCM supernatant CPT-1β activity. *n* = 5–6. (**O**) Quantitative real-time PCR analysis of *Cpt1b* mRNA expression in NRCMs. *n* = 3–4. (**P**) Western blot analysis of CPT-1β protein levels in NRCMs. *n* = 3. (**Q**) Enzyme-linked immunosorbent assay of acetyl-CoA concentration in supernatant of NRCMs. *n* = 4. (**R**) Representative images treated with CPT-1β siRNA and quantification of surface (μm^2^) in NRCMs analyzed by ImageJ 1.53k. Scale bar: 100 μm, 50 μm. *n* = 3–4. (**S**) Quantitative real-time PCR analysis of *Nppa* and *Nppb* mRNA expression in NRCMs treated with CPT-1β siRNA. *n* = 4. (**T**) Representative images and quantification of immunofluorescence staining for αSMA (green), Col1a1 (red) and DAPI (blue) in primary cultured rat cardiac fibroblasts. Merged images are shown. Scale bar: 50 μm. *n* = 3. (**U**) Enzyme-linked immunosorbent assay of acetyl-CoA concentration in supernatant of NRCMs. *n* = 4–6. Data are mean ± SD, * *p* < 0.05, ** *p* < 0.01. ns: no significant difference.

CPT-1β is the rate-limiting enzyme responsible for β-oxidation of long-chain fatty acids [14,15,16,17,18,19]. To explore whether IMD alleviated DCM depended on the role of CPT-1β; we firstly performed RNA sequencing in the hearts of HFD-fed WT mice. HFD-fed WT mice showed 496 up-regulated genes and 367 down-regulated genes (with at least a two-fold change) and significant enrichment in fatty acid metabolism pathways (Figure 4A,B). The heat map revealed the up-regulation of *Cpt1b* in HFD-fed WT mice (Figure 4C). Real-time PCR also revealed the up-regulation of the *Cpt1b* mRNA level in HFD-fed WT mice and in the hearts of fructose-drinking rats (Figure 4D). These results indicated that CPT-1β was a key regulator involved in DCM. Then, we performed RNA sequencing on hearts from HFD-fed, IMD^−/−^ mice and WT mice to explore whether IMD alleviated DCM by regulating CPT-1β. It was found that HFD-fed, IMD^−/−^ mice showed 1397 up-regulated genes and 893 down-regulated genes versus HFD-fed WT mice (with at least a two-fold change) and significant enrichment in fatty acid metabolism pathways (Figure 4E,F). The heat map and real-time PCR showed the down-regulation of *Cpt1b* mRNA expression in HFD-fed, IMD^−/−^ mice (Figure 4G,H). Thus, CPT-1β might be a crucial target for IMD to protect against DCM. Then, we performed RNA sequencing on hearts from HFD-fed, IMDtg mice and WT mice to confirm whether CPT-1β was a key target of IMD. The heat map and real-time PCR showed the up-regulation of *Cpt1b* mRNA expression in HFD-fed, IMDtg mice versus HFD-fed WT mice (Figure 4I,J). Therefore, IMD could up-regulate *Cpt1b* mRNA expression in the myocardium of mice. Then, we further verified whether IMD played a protective role in DCM by up-regulating CPT-1β. CPT-1β is the main isoform in the heart [14,16]. We found that the mRNA level of *Cpt1b* was higher than *Cpt1a* in NRCM (Figure 5A). The enzyme activity of CPT-1β and content of acetyl-CoA generated by fatty acid β-oxidation in plasma were significantly increased in T2DM patients (Figure 5B,C). Fructose-drinking rats showed increased enzyme activity, mRNA, protein levels of CPT-1β, and content of acetyl-CoA, and these levels were further enhanced by IMD administration (Figure 5D–G). Moreover, IMD overexpression elevated the enzyme activity and protein level of CPT-1β and the content of acetyl-CoA in HFD-fed, IMDtg mice (Figure 5H–J), whereas IMD deficiency decreased the above phenomena (Figure 5K–M). 

Similar results were observed in vitro. IMD treatment increased enzyme activity, mRNA, protein levels of CPT-1β, and content of acetyl-CoA in PA-treated NRCMs (Figure 5N–Q). To verify that IMD alleviated DCM via up-regulating CPT-1β, small-interfering RNA (siRNA) of CPT-1β was used to knock down CPT-1β expression in NRCMs. CPT-1β silencing (Figure S4A,B) abolished the effects of IMD in alleviating cardiomyocyte hypertrophy and cardiac fibroblast activation and increasing acetyl-CoA content in NRCMs (Figure 5R–U). These results suggested that IMD alleviated cardiomyocyte hypertrophy and cardiac fibroblast activation in rats by up-regulating CPT-1β.

### 2.6. IMD Up-Regulates CPT-1β via the PI3K/Akt Signaling Pathway

**Figure 6:**
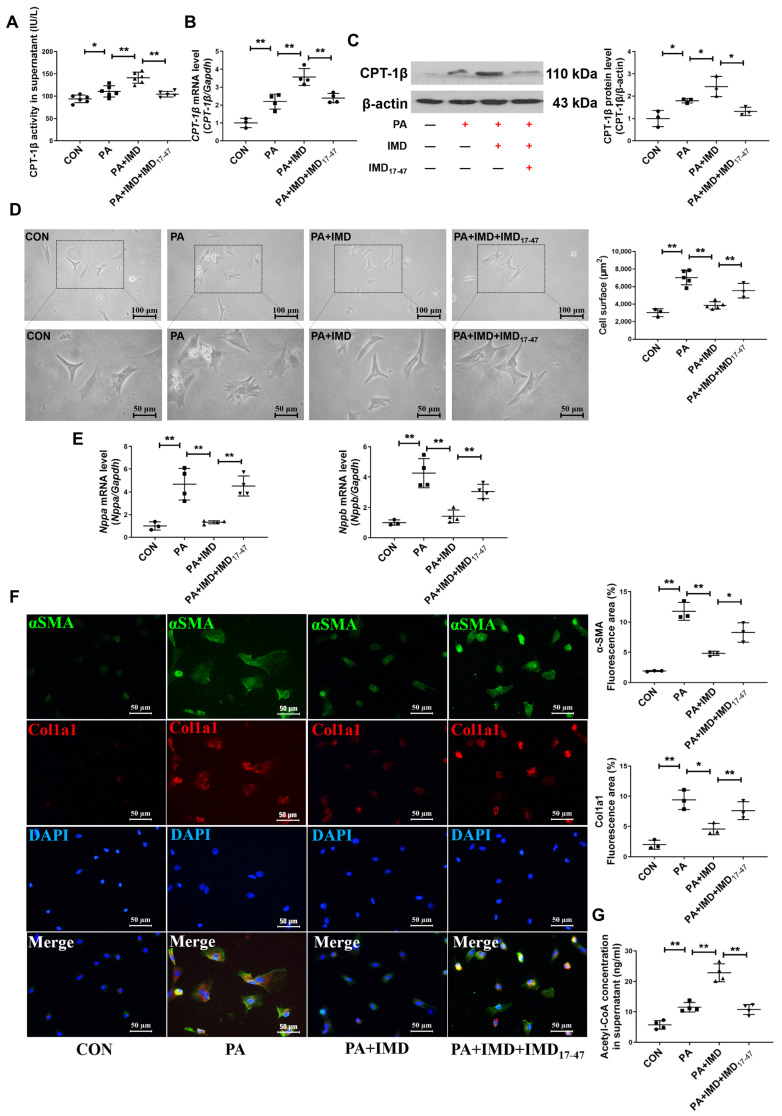
IMD up-regulates CPT-1β via its receptor complex. (**A**) Enzyme-linked immunosorbent assay of NRCM supernatant CPT-1β activity. *n* = 5–6. (**B**) Quantitative real-time PCR analysis of *Cpt1b* mRNA expression in NRCMs. *n* = 3–4. (**C**) Western blot analysis of CPT-1β protein levels in NRCMs. *n* = 3. (**D**) Representative images treated with IMD_17–47_ and quantification of surface (μm^2^) in NRCMs analyzed by ImageJ 1.53k. Scale bar: 100 μm, 50 μm. *n* = 3–5. (**E**) Quantitative real-time PCR analysis of *Nppa* and *Nppb* mRNA expression in NRCMs. *n* = 3–4. (**F**) Representative images and quantification of immunofluorescence staining for αSMA (green), Col1a1 (red) and DAPI (blue) in primary cultured rat cardiac fibroblasts. Merged images are shown. Scale bar: 50 μm. *n* = 3. (**G**) Enzyme-linked immunosorbent assay of acetyl-CoA concentration in the supernatants of NRCMs. *n* = 4. +: added. −: not added. Data are mean ± SD, * *p* < 0.05, ** *p* < 0.01.

IMD binds to its receptor complexes, CRLR/RAMPs, to exert biological effects [30,31,32,33,45]. Firstly, to explore whether IMD alleviated DCM by up-regulating CPT-1β through its receptor complexes, IMD receptor antagonist IMD_17–47_ was used. Preincubation with IMD_17–47_ blocked the effects of IMD on up-regulating enzyme activity, mRNA, and protein levels of CPT-1β in NRCMs treated with PA (Figure 6A–C). Pretreatment with IMD_17–47_ also blocked the effects of IMD on inhibiting cardiomyocyte hypertrophy (Figure 6D,E) and cardiac fibroblast activation (Figure 6F) and increasing acetyl-CoA content (Figure 6G) in PA-treated NRCMs. These results suggested that IMD alleviated cardiomyocyte hypertrophy and cardiac fibroblast activation and increased acetyl-CoA content by up-regulating CPT-1β through its receptor complexes.

**Figure 7:**
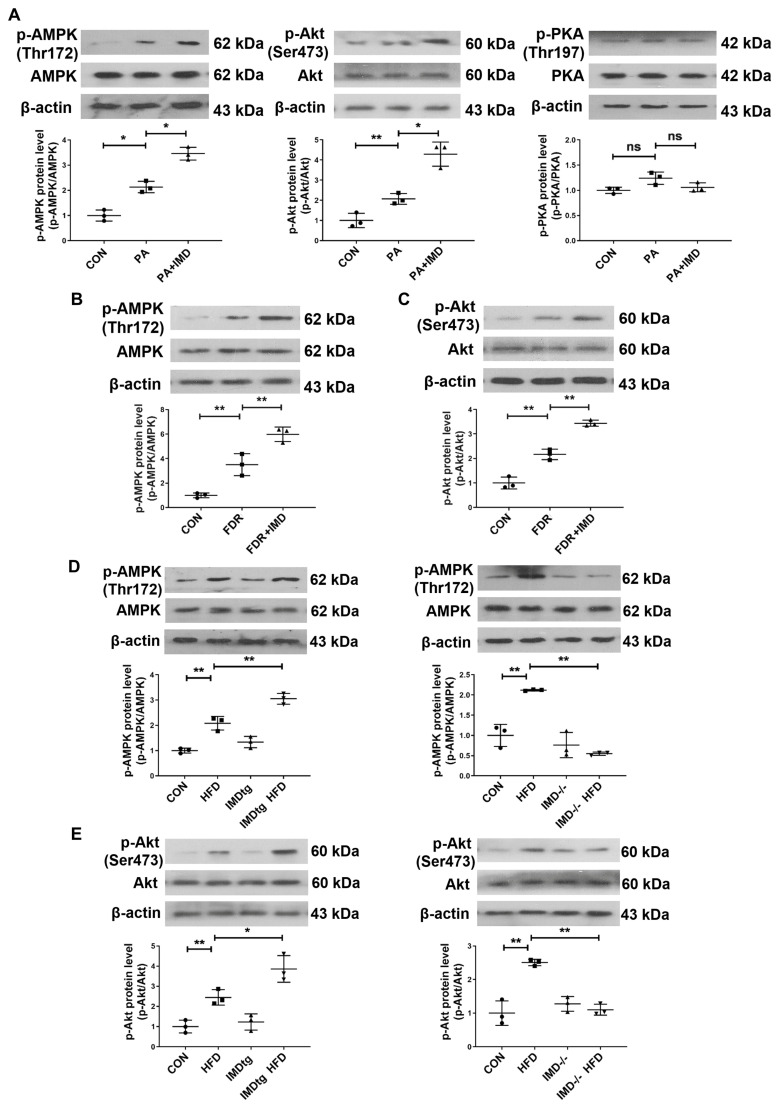

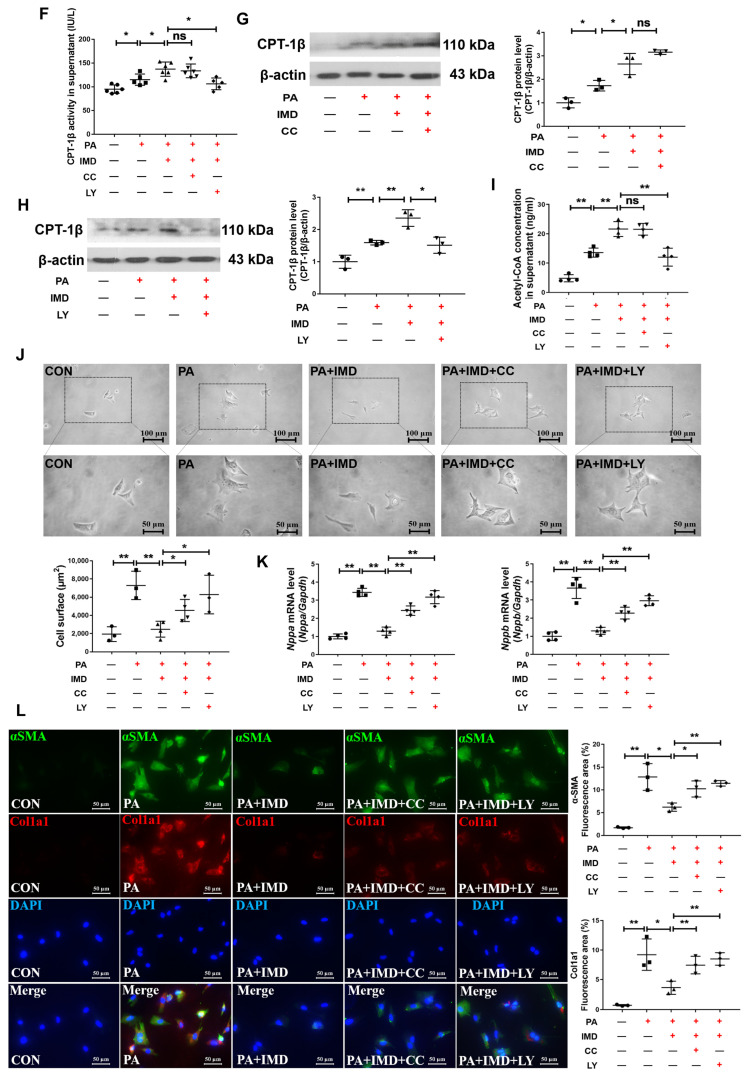
IMD up-regulates CPT-1β via the PI3K/Akt signaling pathway. (**A**) Western blot analysis of p-AMPK (Thr172), AMPK, p-Akt (Ser473), Akt, p-PKA (Thr197), and PKA protein levels in NRCMs. *n* = 3. (**B**) Western blot analysis of p-AMPK (Thr172) and AMPK protein levels in DCM rat hearts. *n* = 3. (**C**) Western blot analysis of p-Akt (Ser473) and Akt protein levels in DCM rat hearts. *n* = 3. (**D**) Western blot analysis of p-AMPK (Thr172) and AMPK protein levels in the hearts of diabetic WT, IMDtg, and IMD^−/−^ mice. *n* = 3. (**E**) Western blot analysis of p-Akt (Ser473) and Akt protein levels in the hearts of diabetic WT, IMDtg, and IMD^−/−^ mice. *n* = 3. (**F**) Enzyme-linked immunosorbent assay of NRCM supernatant CPT-1β activity. *n* = 5–6. (**G**,**H**) Western blot analysis of CPT-1β protein levels in NRCMs. *n* = 3. (**I**) Enzyme-linked immunosorbent assay of acetyl-CoA concentration in the supernatants of NRCMs. *n* = 4. (**J**) Representative images and quantification of surface (μm^2^) in NRCMs analyzed by ImageJ 1.53k. Scale bar: 100 μm, 50 μm. *n* = 3–4. (**K**) Quantitative real-time PCR analysis of *Nppa* and *Nppb* mRNA expression in NRCMs. *n* = 4. (**L**) Representative images and quantification of immunofluorescence staining for αSMA (green), Col1a1 (red) and DAPI (blue) in primary cultured rat cardiac fibroblasts. Merged images are shown. Scale bar: 50 μm. *n* = 3. +: added. −: not added. Data are mean ± SD, * *p* < 0.05, ** *p* < 0.01. ns: no significant difference.

The binding of IMD to its receptor complexes leads to the activation of post-receptor signaling pathways, such as cyclic adenosine monophosphate/protein kinase A (cAMP/PKA), AMP-activated protein kinase (AMPK), and PI3K/Akt pathways [30,31,32]. In this study, we found that the phosphorylation of AMPK and Akt was apparently elevated in PA-treated NRCMs and heart tissues of fructose-drinking rats, which were further enhanced by IMD administration, whereas IMD treatment had no significant effect on the phosphorylation of PKA in PA-treated NRCMs (Figure 7A–C). IMD overexpression elevated the phosphorylation of AMPK and Akt in the heart tissues of HFD-fed, IMDtg mice, whereas IMD deficiency decreased the above phenomena (Figure 7D,E). These findings indicated that AMPK and PI3K/Akt signaling pathways were activated by IMD in DCM and hypertrophic cardiomyocytes. To verify whether AMPK and PI3K/Akt mediated the effects of IMD on up-regulating CPT-1β, AMPK inhibitor Compound C and PI3K inhibitor LY294002 were used. Only LY294002 pretreatment blocked the effects of IMD on up-regulating enzyme activity and the protein level of CPT-1β and the content of acetyl-CoA in NRCMs (Figure 7F–I), although both Compound C and LY294002 reversed the effects of IMD on alleviating cardiomyocyte hypertrophy and cardiac fibroblast activation in rats (Figure 7J–L). These results indicated that IMD up-regulated CPT-1β, then inhibited cardiomyocyte hypertrophy and cardiac fibroblast activation, and increased the content of the acetyl-CoA via PI3K/Akt signaling pathway. 

## 3. Discussion

In this study, we highlighted the beneficial effect of IMD on DCM and its underlying mechanisms. Specifically, this study innovatively demonstrated that (a) the levels of IMD and its receptor complexes were reduced in the heart tissues of DCM rats and mice. (b) Exogenous and endogenous IMD mitigated cardiac hypertrophy, fibrosis, dysfunction, and lipid accumulation in DCM rats and IMD-transgenic DCM mice, whereas knockout of IMD worsened these pathological processes in IMD-knockout DCM mice. (c) CPT-1β enzyme activity, mRNA and protein levels, and acetyl-CoA content were increased in T2DM patients, rats and mice. IMD increased enzyme activity, mRNA, protein levels of CPT-1β, and the content of acetyl-CoA in the serum and cardiac tissues of DCM rats. IMD overexpression elevated the enzyme activity and protein level of CPT-1β and the content of acetyl-CoA in serum and cardiac tissues of IMDtg DCM mice. Knockdown of CPT-1β blocked the effects of IMD on increasing the acetyl-CoA content in PA-treated NRCMs and on inhibiting cardiomyocyte hypertrophy and cardiac fibroblast activation. IMD receptor antagonist IMD_17–47_ and PI3K/Akt inhibitor LY294002 reversed the effects of IMD on up-regulating enzyme activity, mRNA, and protein level of CPT-1β and on the content of acetyl-CoA and on inhibiting cardiomyocyte hypertrophy and cardiac fibroblast activation. We revealed that IMD alleviates DCM by up-regulating CPT-1β via CRLR/RAMP receptor complexes and PI3K/Akt signaling. Therefore, targeting the restoration of the IMD level may be a potential clinical therapeutic target for DCM treatment.

Our study revealed the protective role of IMD in DCM. To explore the effect of IMD on DCM, rats and wildtype, IMD^−/−^, and IMDtg mice were used [8,39,46,47,48,49,50,51] to induce DCM under different pathologies. We did not observe any abnormalities in IMDtg mice and IMD^−/−^ mice, which are consistent with previous studies [34,40,41,51,52]. In this study, IMDtg mice and IMD^−/−^ mice did not have cardiac hypertrophy, fibrosis, dysfunction, hypertension, and abnormal glucose and lipid metabolism, which were indistinguishable from their WT littermates. We did not observe other systems or organs of IMDtg mice and IMD^−/−^ mice; this is a limitation. In other studies, IMD^−/−^ mice exhibited a primary immunosuppression phenotype, impaired vessel hierarchical structure, reduced blood perfusion, vascular leakage, and increased inflammation [53,54,55,56]. Therefore, IMD plays an important role in vascular structure, vascular development, and inflammatory immunity.

According to previous studies, with minor modifications [57,58], drinking 10% fructose water for 12 weeks resulted in insulin resistance, cardiac hypertrophy, and fibrosis in SD rats. HFD-fed resulted in insulin resistance, cardiac hypertrophy, fibrosis and diastolic and systolic dysfunction and lipid accumulation in mice [46,47,48,49,50,51]. 

Endogenous IMD is found in the brain, pituitary, heart, kidney, gastrointestinal tract, plasma, pancreas, lung, spleen, thymus, and ovary [33]. Endogenous IMD is highly expressed in the heart, which can secrete from cardiomyocytes and cardiac fibroblasts [30,31,32,33,34,36,59]. In this study, we did not detect the expression of IMD in cardiac fibroblasts, which is a limitation. In this study, we found that cardiac mRNA levels of *IMD*, mRNA, and protein levels of its receptor complexes, CRLR/RAMPs, were reduced in DCM rats and mice, which is consistent with previous studies [60]. Other studies reported that IMD expression decreased in the plasma, cardiac tissue, adipose tissue, and aortic root atherosclerotic lesions of db/db mice, HFD-induced obese mice, hyperhomocysteinemia mice, HFD-fed ApoE-/- mice, and diabetic rats [40,41,42,51,60]. CGRP superfamily members ADM and CGRP are down-regulated in the myocardium of diabetic rats [61,62]. We also detected that mRNA levels of IMD were down-regulated in hypertrophic cardiomyocytes. These data may indicate that IMD acts in an autocrine or paracrine manner [40]. T2DM characterized with hyperglycemia due to impaired insulin secretion and/or action may lead to the down-regulation of IMD, indicating that IMD is involved in DCM [2]. Hirose et al. reported that the expression of AM2/IMD is enhanced in the failing heart, and up-regulated AM2/IMD in the failing heart seems to be related to its cardio-protective effects [38,63]. In this study, although IMD is down-regulated in DCM, exogenous IMD administration protected against DCM in T2DM rats. These differences might be attributed to different disease states and stages leading to different expression levels of IMD. We found that CPT-1β enzyme activity, mRNA, and protein levels were increased in T2DM patients, rats and mice, which is in line with Nakamura’s report [17]. These results suggested that decreased endogenous IMD led to compensatory up-regulation of CPT-1β. 

Then, we observed that both exogenous IMD administration and IMD overexpression mitigated cardiac hypertrophy, fibrosis, diastolic and systolic dysfunction, and lipid accumulation in T2DM rats and mice. IMD alleviated cardiomyocyte hypertrophy and cardiac fibroblast activation in rats induced by PA. Conversely, IMD deficiency exacerbated the development of DCM in T2DM mice. These data revealed that IMD was essential for alleviating DCM. In line with previous studies [30,34,35,36,37,38,40,41,59], IMD can improve many cardiovascular diseases such as diabetic ischemic heart injury, cardiac hypertrophy, cardiac fibrosis, congestive heart failure, and hypertension. IMD also can improve metabolic syndromes such as obesity and insulin resistance in T1DM rats, T2DM mice, and ApoE^−/−^ atherosclerosis mice [39,40,41,42]. 

CPT-1β is the rate-limiting enzyme responsible for β-oxidation of long-chain fatty acids [14,15,16,17,18,19]. Overexpression of CPT-1 can enhance fatty acid oxidation, reduce lipid accumulation, and improve insulin sensitivity in HFD-fed rats [18]. Deletion or inhibition of CPT-1β can lead to the inhibition of fatty acid oxidation, myocardial lipid accumulation, impaired insulin sensitivity, heart regeneration, cardiac hypertrophy, cardiac fibrosis, and cardiac dysfunction in mice [14,19,20,21,22,23]. Moreover, many studies have reported that multiple bioactive molecules regulate CPT-1β expression [24,25,26,27]. In this study, we found that IMD up-regulated the CPT-1β mRNA level, protein level and enzyme activity, and acetyl-CoA content in T2DM rats, T2DM mice, and hypertrophic cardiomyocytes. Then, CPT-1β knockdown prevented the effects of IMD on inhibiting cardiomyocyte hypertrophy, cardiac fibroblast activation, and up-regulating acetyl-CoA content. Thus, these findings revealed that IMD may protect against DCM by up-regulating CPT-1β expression. 

IMD binds to its receptor complexes, CRLR/RAMPs, and activates post-receptor signaling pathways such as cAMP/PKA, AMPK, and PI3K/Akt to exert biological effects [30,31,32]. In this work, we found that the phosphorylation of AMPK and Akt was apparently elevated in cardiac tissues of DCM rats and mice and in hypertrophic cardiomyocytes, which were further enhanced after exogenous IMD administration and IMD overexpression. Only PI3K inhibitor LY294002 pretreatment blocked the effects of IMD on up-regulating CPT-1β enzyme activity and protein levels and acetyl-CoA content in NRCMs. These results indicated that IMD up-regulated CPT-1β and then inhibited PA-induced rat cardiomyocyte hypertrophy and cardiac fibroblast activation and increased the NRCM content of acetyl-CoA via the PI3K/Akt signaling pathway. These phenomena were in line with previous studies [64,65,66,67,68]. However, our results showed that the inhibition of AMPK did not block the up-regulatory effect of IMD on CPT-1β. In this study, we found that IMD alleviates DCM through activation of the PI3K/Akt signaling pathway. PI3K/Akt can be activated by phosphorylation of the insulin receptor substrate after insulin binding to its receptor [10,64,69]. Insulin can alleviate DCM by binding to its receptor located at the plasma membrane and then stimulating the uptake of glucose into cardiac muscle [10,69,70,71,72,73,74]. In this study, we found that fasting serum insulin levels increased, and insulin resistance was observed in T2DM rats and mice, which are consistent with previous studies [10,51,70]. Tissue resistance to the action of insulin constitutes the hallmark of T2DM, promoting reduced insulin sensitivity and compensatory increase in insulin [10,51,70]. It was reported that IMD can protect against diabetic ischemic heart injury in rats [59]. CGRP superfamily members ADM and CGRP can also improve cardiac dysfunction in diabetic rats [66,75]. In this study, we found that IMD can alleviate DCM by up-regulating CPT-1β through activation of the PI3K/Akt signaling pathway, which is consistent with previous studies [59,66,75]. IMD might also alleviate DCM partly through improving insulin resistance in T2DM rats and mice.

## 4. Materials and Methods

### 4.1. Human Subjects 

Between January 2017 and December 2017, male participants with type 2 diabetes mellitus (*n* = 38) and without diabetes (*n* = 41) were included in this study. Inclusion criteria and exclusion criteria were previously described [51]. Informed consent was obtained from all participants for this study. The study on humans was conducted according to the ethical guidelines of the 1975 Declaration of Helsinki. The protocol was approved by the Ethics Committee of Peking University International Hospital (No. 2016-064).

### 4.2. Animals

All animal care and experimental protocols followed the Guide for the Care and Use of Laboratory Animals published by the US National Institutes of Health (NIH Publication, 8th Edition, 2011). The Animal Care Committee of Peking University Health Science Center (Beijing, China) approved all animal experimental procedures (No. LA2020042). All experiments complied with international guidelines on the ethical use of animals.

Sprague Dawley (SD) rats and Neonatal SD rats (1–3-day-old) were purchased from the Animal Center, Peking University Health Science Center (No. LA2020042). The Model Animal Research Center of Nanjing University (Nanjing, China) provided IMDtg mice, IMD^−/−^ mice, and their WT littermates as controls with C57BL/6 background, as described before [59,76]. 

### 4.3. DCM Models of Rats and Mice 

Male SD rats (8 weeks old) were randomly assigned to 3 groups: (1) control (CON) group—rats were given normal drinking water for 12 weeks; (2) fructose-drinking rat (FDR) group—rats were given 10% fructose drinking water for 12 weeks; and (3) FDR plus IMD (FDR + IMD) group—rats were given 10% fructose drinking water for 12 weeks and 4 weeks of subcutaneous infusion of IMD at 100 ng/kg/h via an Alzet Mini-osmotic Pump [57,58] with minor modifications. All rats were fasted overnight and killed by exsanguination. The hearts were rapidly removed for further studies. 

Male IMD^−/−^, IMDtg, and WT littermate mice (8–10 weeks old) were randomly assigned to 2 groups: (1) CON group—mice received a normal diet for 16 weeks and (2) HFD group—mice were fed with HFD (60% kcal from lard, D12492) for 16 weeks [46,47,48]. After echocardiography and hemodynamic measurements, all mice were fasted overnight and killed by exsanguination. The hearts were rapidly removed for further studies.

### 4.4. Echocardiography

Mice were subjected to echocardiography using a Vevo 770 T M Imaging System (Visual Sonics, Toronto, ON, Canada) and a 30 MHz probe. We used 2-D-directed M-mode from the LV short axis to record the LV internal diameter in systole (LVID; s) or in diastole (LVID; d), LV posterior wall thickness in systole (LVPW; s) or in diastole (LVPW; d), LV anterior wall thickness in systole (LVAW; s) or in diastole (LVAW; d), LV volume in systole (LV Vol; s) or in diastole (LV Vol; d), LV EF, and FS. Early diastolic mitral flow velocity (E)/late diastolic mitral flow velocity (A) was evaluated by pulse Doppler ultrasound. Data from 5 consecutive cardiac cycles were averaged. 

### 4.5. Blood Pressure Measurement

After echocardiography, the non-invasive computerized tail-cuff system (BP-98A, Softron, Tokyo, Japan) was used to measure the systolic blood pressure (SBP), diastolic blood pressure (DBP), and mean blood pressure (MBP) of the mice. A thermostat was used to keep the body temperature of the mice constant before blood pressure was measured (30 °C–33 °C). The blood pressure of the mice was monitored continuously until the blood pressure of the mice stabilized. Data from 5 individual measurements were averaged [34].

### 4.6. Neonatal Rat Cardiomyocyte (NRCM) and Cardiac Fibroblast Culture

We isolated ventricles from neonatal SD rats (1–3 days old), shredded and digested them, and collected the supernatant. The cell suspension was filtered and pre-plated in two 25-mm^2^ culture flasks for 90 min at 37 °C to allow the fibroblasts to adhere to the flasks. The non-attached cardiomyocytes were plated in several 6-well cell culture plates. Cardiomyocytes exhibited a rhombic, triangular, or polygonal shape under the microscope. The cardiomyocytes formed beating islands. Fibroblasts were spindle-shaped or star-like (Figure S5A). Fibroblast purity was assessed by staining with Vimentin (Figure S5B).

Then, 300 μmol/L PA was selected as the optimal concentration and 24 h was selected as the optimal stimulation time based on the effect on cell viability (Figure S6A–D) [77,78]. The cardiomyocytes and fibroblasts were starved overnight with a 1% fetal bovine serum medium. After incubation with IMD (1 × 10^−7^ mol/L; Phoenix Pharmaceuticals, Belmont, CA, USA) for 30 min, the cardiomyocytes and fibroblasts were stimulated with PA (300 μmol/L) for 24 h to induce cardiomyocyte hypertrophy and fibroblast activation [34,35,77,78,79,80]. To explore the role of CRLR/RAMPs and their signaling pathway, the cardiomyocytes and fibroblasts were pre-incubated for 30 min with IMD receptor antagonist IMD_17–47_ (10^−6^ mol/L; Phoenix Pharmaceuticals), PI3K inhibitor LY294002 (10 μmol/L; Sigma, St. Louis, MO, USA), and AMPK inhibitor Compound C (10 μmol/L; Sigma) [51] prior to IMD and PA treatment.

### 4.7. H&E, Sirius Red, and Oil Red O Staining

The hearts were fixed for 10–12 h, embedded in paraffin and sectioned (5 μm), and stained with H&E, as previously described [34]. Other sections were stained with a 0.1% Sirius-red picrate solution for 60 min. The frozen slides were covered with Oil Red O working solution for 30 min. Images were acquired using a microscope. The stained ventricular area was analyzed with ImageJ 1.53k software to quantify cardiac fibrosis and the cardiac myocyte cross-sectional area [34].

### 4.8. Immunofluorescence Staining of Cardiac Fibroblast

Cardiac fibroblasts were fixed for 10 min and washed with PBS. The cells were incubated with antibodies for α-SMA (1:100) and Col1a1 (1:100) at 4 °C overnight. Then, cells were incubated with fluorescein-labeled secondary antibody (Millbrae, CA, USA). Nuclei were counterstained with 4′,6-diamidino-2-phenylindole (DAPI) (Invitrogen, Carlsbad, CA, USA). Immunofluorescent images were acquired using a Leica Imaging Systems microscope (Cambridge, UK).

### 4.9. Detection of Fasting Serum Glucose and Insulin and Homeostasis Model Assessment for Insulin Resistance (HOMA-IR) Analysis

Mouse and rat serum were obtained from blood samples, which were precipitated under natural static conditions. FBG levels of rats and mice were determined with a detection kit (100000240, Zhong Sheng Biotechnology, Beijing, China). The enzyme-linked immunosorbent assay (ELISA) kit, in accordance with the manufacturer’s instructions (E-EL-R3034, E-EL-M1382c, Elabscience, Wuhan, China), were used to determine the serum insulin levels of the rats and mice. The HOMA-IR was calculated as follows, HOMA-IR = [Insulin (μIU/mL) × Blood glucose (mmol/L)]/22.5 [81].

### 4.10. Quantitation of Serum Lipids in Mice and Rats

Serum from the rats and mice was obtained from blood samples, which were precipitated under natural static conditions at the end of the experiment. Total cholesterol (100000180), triglycerides (100000220), low-density lipoprotein cholesterol (LDL-C) (100020245), and high-density lipoprotein cholesterol (HDL-C) (100020235) detection kits were from Zhong Sheng Biotechnology (Beijing, China) and were used to determine lipid profiles. The serum of the rats and mice were mixed separately with working fluid, and OD values were detected by a biochemical analyzer to determine the levels of total cholesterol, triglycerides, LDL-C, and HDL-C.

### 4.11. ELISA 

Rat and mouse serums were obtained from blood samples, which were precipitated in natural static conditions at the end of the experiment. Cardiomyocytes were stimulated with PA after incubation with IMD. The ELISA kits for mouse, rat, and human CPT-1β (MM-46720M2, MM-71573R2, MM-61760H1) were purchased from Jiangsu Meimian (Yancheng, China). The ELISA kits for mouse, rat, and human acetyl-CoA (ER11934, ER07786, ER01745) were purchased from Zancheng (Tianjin, China). We compared the O.D. to the standard curve from the manufacturer’s instructions to determine CPT-1β enzymatic activity and acetyl-CoA content in rat and mouse serum, human plasma, and supernatant of the cardiomyocytes.

### 4.12. Western Blot Analysis

Equal amounts of total protein from heart tissues or cell extracts were resolved by 10% or 12% SDS-PAGE. We incubated the blots with primary antibodies β-actin (sc-47778, Santa Cruz, CA, USA, 1:2000), CRLR (GTX64616, GeneTex, Irvine, CA, USA, 1:500), RAMP1 (ab156575, Abcam, Cambridge, UK, 1:1000), RAMP2 (sc-365240, 1:300), RAMP3 (sc-365313, 1:300), Col1a1 (ab260043, 1:500), Col3a1 (22734-1-AP, Proteintech, Chicago, IL, USA, 1:500), CPT-1β (A6796, ABclonal, Wuhan, China, 1:1000), p-AMPK (Thr172) (2531, Cell Signaling Technology, Danvers, MA, USA, 1:1000), AMPK (2532, Cell Signaling Technology, 1:1000), p-Akt (Ser473) (4060, Cell Signaling Technology, 1:500), Akt (9272, Cell Signaling Technology, 1:1000), p-PKA (Thr197) (sc-32968, 1:1000), and PKA (sc-32968, 1:1000) overnight, then with secondary antibodies for 1 h. Protein levels were analyzed with NIH ImageJ 1.53k and normalized to β-actin.

### 4.13. Quantitative Real-Time PCR Analysis

Total RNA from cardiac tissue or cultured cardiomyocytes was extracted with Trizol. The RNA-to-cDNA kit (Novoprotein, Shanghai, China) was used to convert RNA to cDNA. The Applied Biosystems 7500 fast PCR System (Life Technologies, Carlsbad, CA, USA) and SYBR Green I reagent (Novoprotein, Shanghai, China) were used for quantitative real-time PCR amplification. Primer sequences for real-time PCR is shown in Table S8. The cycle threshold (Ct) reflected the number of PCR cycles required for the fluorescent signal to cross a set threshold. The 2^−ΔΔCt^ method was used for relative quantification, with *Gapdh* as a reference. 

### 4.14. RNA Sequencing 

RNA extracted from the hearts of three diabetic WT, IMDtg and IMD^−/−^ mice were used for RNA sequencing. Fresh heart tissues were obtained right after killing the mice. We then quickly placed the heart tissues in liquid nitrogen. The qualified total RNA was extracted, purified, and reverse-transcribed after fragmentation, and the cDNA library was constructed. RNA samples (1 μg) were used for library preparation using the NEBNext Ultra RNA sample preparation kit (Illumina). Differential gene expression analysis was estimated in the DESeq2 R package (1.16.1). Genes with an adjusted *p* value < 0.05 were assigned as differentially expressed. 

### 4.15. siRNA Transfection and Identification

CPT-1β siRNAs (siCPT-1β) and negative scramble siRNA were from RiboBio (Guangzhou, China). Using the Lipofectamine RNAiMAX Transfection Reagent (Invitrogen, Carlsbad, CA, USA), we incubated NRCMs at 50% confluence with CPT-1β siRNA or negative control siRNA at a final concentration of 50 nmol for 6 h. After transfecting with serum-free Dulbecco’s modified Eagle medium for 6 h, we changed the medium to a serum-containing medium. Quantitative RT-PCR and Western blot analysis were used to evaluate gene knockdown efficiency after transfection at 36 h and 72 h, respectively.

### 4.16. Statistical Analysis

All the analyses were performed using GraphPad Prism version 7.0 (GraphPad Software Inc., San Diego, CA, USA). Normal distribution was tested using the Shapiro–Wilk test. Homogeneity of variances was examined using the Brown–Forsythe test. All data were expressed as the mean  ±  SD. Student’s t-test was used to compare two groups, and one-way ANOVA or two-way ANOVA, followed by Tukey’s post hoc test, were used for multiple group comparisons. A *p*-value of less than 0.05 indicated a statistically significant difference.

## 5. Conclusions

In conclusion, our study revealed the protective role of the cardiovascular bioactive peptide IMD in DCM. IMD may attenuate DCM by up-regulating CPT-1β via CRLR/RAMP receptor complexes and PI3K/Akt signaling. Targeting IMD as a therapeutic strategy could have potential for DCM prevention and treatment.

## Data Availability

The data presented in this study are contained within the article.

## Supplementary Materials

The following supporting information can be downloaded at: https://www.mdpi.com/article/10.3390/ph17091204/s1, Figure S1. IMD and IMD receptor levels are decreased in the hearts of diabetic rats. Figure S2. IMD and IMD receptor levels are decreased in the hearts of diabetic mice. Figure S3. Verify the levels of IMD in the hearts of IMDtg mice and IMD^−/−^ mice. Figure S4. Expression of CPT-1β after administration of CPT-1β siRNA in NRCMs. Figure S5. The identification of NRCMs and cardiac fibroblasts. Figure S6. Effects of different concentrations and different treatment times of palmitic acid on the viability of NRCMs and cardiac fibroblasts. Table S1. Glucose and lipid metabolic data of diabetic rats. Table S2. Echocardiographic parameters of diabetic WT and IMDtg mice. Table S3. Blood pressure in diabetic WT and IMDtg mice. Table S4. Glucose and lipid metabolic data of diabetic WT and IMDtg mice. Table S5. Echocardiographic parameters of diabetic WT and IMD^−/−^ mice. Table S6. Blood pressure in diabetic WT and IMD^−/−^ mice. Table S7. Glucose and lipid metabolic data of diabetic WT and IMD^−/−^ mice. Table S8. Primer sequences for real-time PCR.
