# Supplementary material for: Intermedin Alleviates Diabetic Cardiomyopathy by Up-Regulating CPT-1β through Activation of the Phosphatidyl Inositol 3 Kinase/Protein Kinase B Signaling Pathway
Source: Pharmaceuticals (Basel). 2024 Sep 12;17(9):1204. doi: 10.3390/ph17091204 (PMC11435185; doi:10.3390/ph17091204)
Supplement: Supplementary file 1 [file pharmaceuticals-17-01204-s001.zip › pharmaceuticals-3093411-supplementary.pdf]

Supplementary Materials

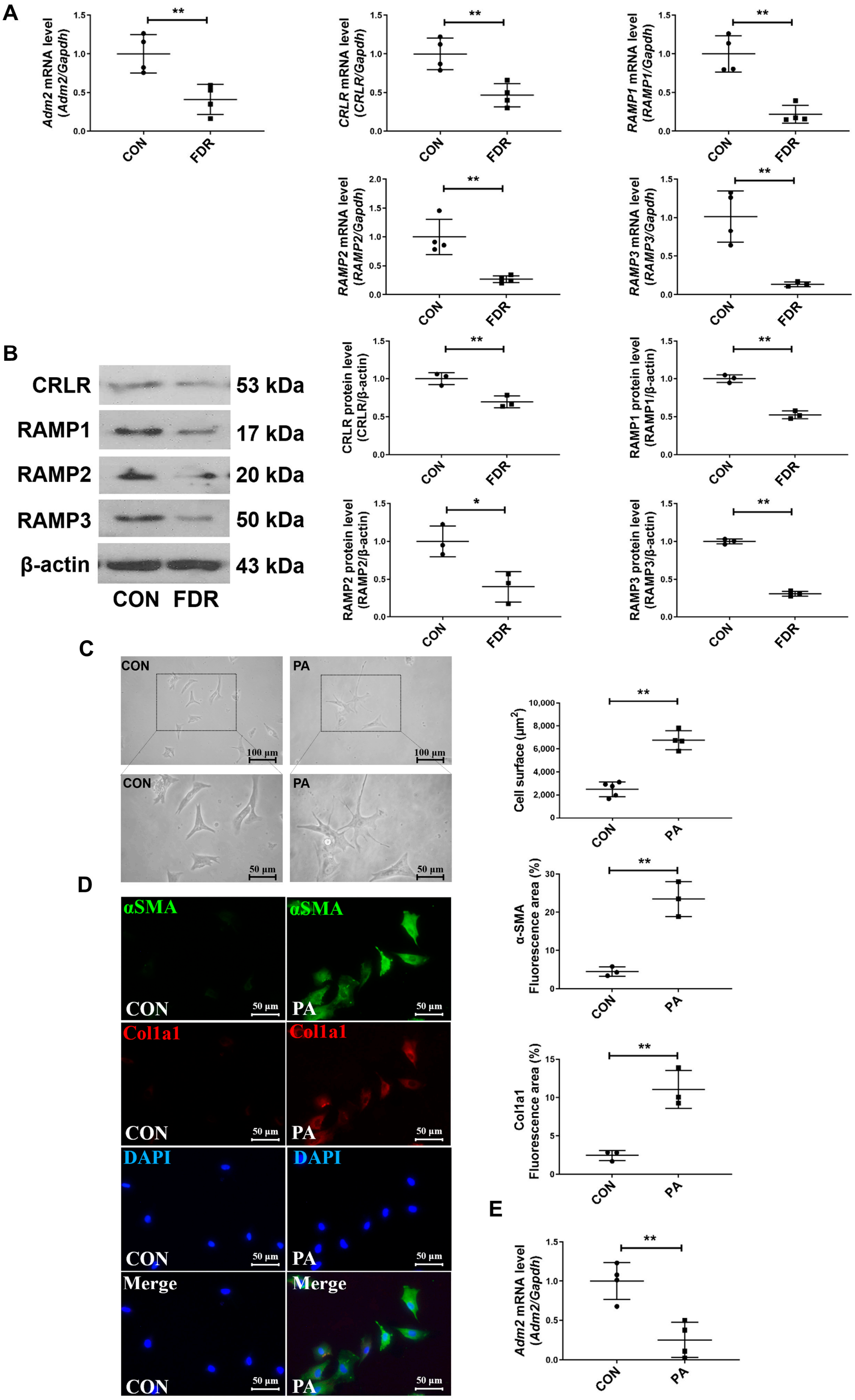

Figure S1. IMD and IMD receptor levels are decreased in the hearts of diabetic rats. (A) Quantitative real-time PCR analysis of mRNA levels of *Adm2*, *CRLR*, *RAMP1*, *RAMP2* and *RAMP3* in DCM rat hearts. n = 3-4. (B) Western blot analysis of protein levels of CRLR，RAMP1，RAMP2，RAMP3 in DCM rat hearts. n = 3. (C) Representative images of NRCMs and quantification of surface

(μm²) analyzed by ImageJ. Scale bar: 100 μm, 50 μm. n = 4-5. (D) Representative images and quantification of immunofluorescence staining for αSMA (green) and Col1a1 (red) in primary cultured rat cardiac fibroblasts. Nuclei were stained with DAPI (blue). Merged images are shown. Scale bar: 50 μm. n = 3. (E) Quantitative real-time PCR analysis of mRNA levels of *Adm2* in NRCMs. n = 4. Data are mean ± SD, *p<0.05, **p<0.01.

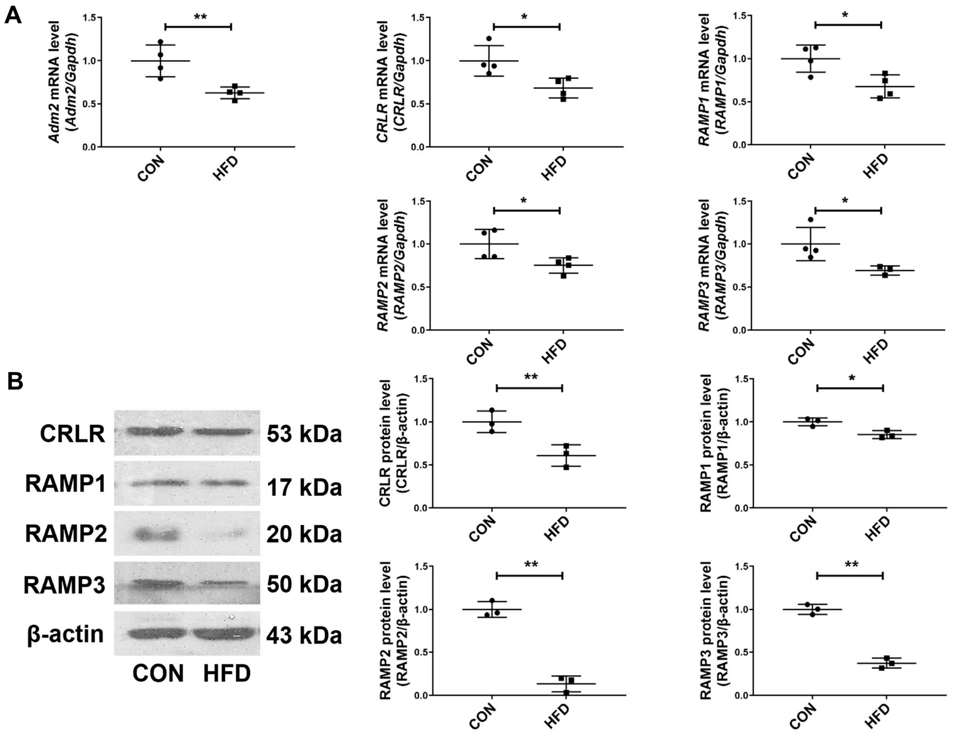

Figure S2. IMD and IMD receptor levels are decreased in the hearts of diabetic mice. (A) Quantitative real-time PCR analysis of mRNA levels of *Adm2*, *CRLR*, *RAMP1*, *RAMP2* and *RAMP3* in the hearts of WT diabetic mice. n = 3. (B) Western blot analysis of protein levels of CRLR，RAMP1，RAMP2，RAMP3 in the hearts of WT diabetic mice. n = 3. Data are mean ± SD, *p<0.05, **p<0.01.

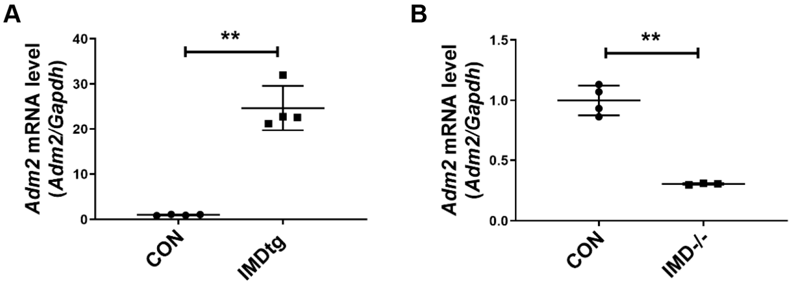

Figure S3. Verify the levels of IMD in the hearts of IMDtg mice and IMD$^{-/-}$ mice. (A) Quantitative real-time PCR analysis of mRNA level of *Adm2* in IMDtg mice. n = 4. (B) Quantitative real-time PCR analysis of mRNA level of *Adm2* in IMD$^{-/-}$ mice. n = 3-4. Results are normalized to level of Gapdh (qPCR). Data are mean ± SD, **p<0.01.

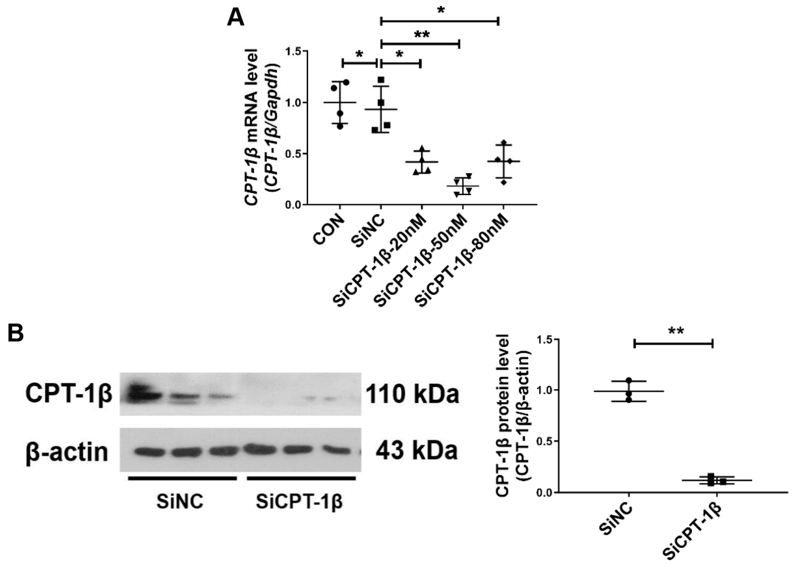

Figure S4. Expression of CPT-1β after administration of *CPT-1β* siRNA in NRCMs. (A) Quantitative real-time PCR analysis of mRNA level of *CPT-1β* in NRCMs. n = 4. (B) Western blot analysis of protein level of CPT-1β in NRCMs. n = 3. Data are mean ± SD, **p*<0.05, ***p*<0.01.

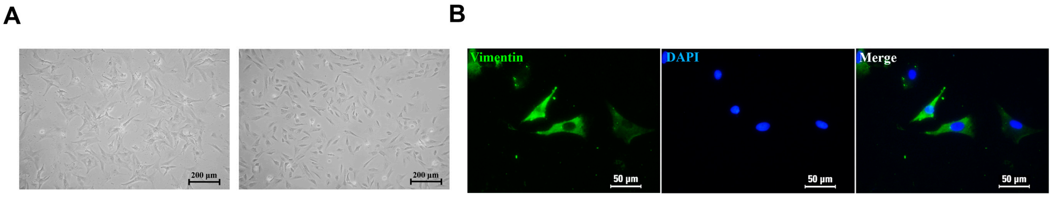

Figure S5. The identification of NRCMs and cardiac fibroblasts. (A) Representative images of NRCMs and cardiac fibroblasts under the microscope. Scale bar: 200 μm. (B) Representative images and quantification of immunofluorescence staining for Vimentin (green) in primary cultured rat cardiac fibroblasts. Nuclei were stained with DAPI (blue). Scale bar: 50 μm.

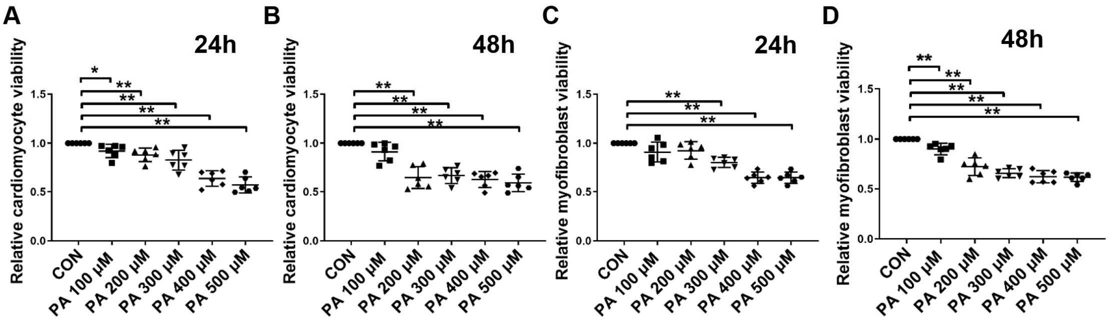

Figure S6. Effects of different concentrations and different treatment times of palmitic acid on the viability of NRCMs and cardiac fibroblasts. (A-D) Effects of different concentrations and different treatment times of palmitic acid on the viability of NRCMs and cardiac fibroblasts. n = 6. Data are mean ± SD, **p*<0.05, ***p*<0.01.

Table S1. Glucose and lipid metabolic data of diabetic rats. FBG, insulin levels, HOMA-IR index, triglycerides, total cholesterol, LDL-C and HDL-C in T2DM rats. n = 6-7. Data are mean ± SD, $^{**}p<0.01$ vs. CON, $^{\#\#}p<0.01$ vs. HFD.

Variables	CON (n = 6-7)	FDR (n = 6-7)	FDR+IMD (n = 6-7)
FBG (mmol/L)	5.95 ± 0.66	11.69 ± 1.76 **	7.87 ± 0.98 ##
Fasting insulin (mIU/L)	10.76 ± 2.38	27.26 ± 1.57 **	12.10 ± 3.17 ##
HOMA-IR	2.85 ± 0.69	14.23 ± 2.66 **	4.21 ± 1.03 ##
Triglycerides (mmol/L)	0.67 ± 0.22	2.37 ± 0.43 **	0.74 ± 0.24 ##
Cholesterol (mmol/L)	1.38 ± 0.55	1.65 ± 0.38	1.59 ± 0.09
LDL-C (mmol/L)	0.44 ± 0.08	0.70 ± 0.14 **	0.44 ± 0.05 ##
HDL-C (mmol/L)	0.43 ± 0.07	0.25 ± 0.05 **	0.37 ± 0.08 ##

Table S2. Echocardiographic parameters of diabetic WT and IMDtg mice. LVID; s, LVPW; s, LVAW; s, LV Vol; s, LVID; d, LVPW; d, LVAW; d, LV Vol; d, EF, FS and LV mass of diabetic WT and IMDtg mice quantified by ultrasonography. n = 5-8. Data are mean ± SD, $^{*}p<0.05$, $^{**}p<0.01$ vs. CON, $^{\#}p<0.05$, $^{\#\#}p<0.01$ vs. HFD.

Variables	CON (n = 5)	HFD (n = 5-8)	IMDtg (n = 5-7)	IMDtg HFD (n = 5-8)
LVID; s (mm)	2.94 ± 0.36	3.57 ± 0.03 *	3.16 ± 0.14	3.24 ± 0.18 ##
LVPW; s (mm)	0.95 ± 0.05	1.39 ± 0.06 **	0.89 ± 0.04	1.05 ± 0.22 #
LVAW; s (mm)	0.92 ± 0.10	1.30 ± 0.13 **	0.83 ± 0.04	0.90 ± 0.10 ##
LV Vol; s (μl)	18.81 ± 0.93	27.04 ± 1.75 **	19.74 ± 1.22	23.37 ± 1.21
LVID; d (mm)	3.83 ± 0.20	4.32 ± 0.10 **	3.87 ± 0.09	3.93 ± 0.17 ##
LVPW; d (mm)	0.98 ± 0.12	1.21 ± 0.04 **	0.97 ± 0.10	1.01 ± 0.14 #
LVAW; d (mm)	0.94 ± 0.06	1.23 ± 0.06 **	0.89 ± 0.10	0.91 ± 0.10 ##
LV Vol; d (μl)	55.66 ± 0.72	63.25 ± 1.82 *	51.35 ± 3.04	57.14 ± 2.51
EF (%)	62.85 ± 1.10	49.10 ± 1.21**	61.94 ± 0.76	56.98 ± 0.97 ##
FS (%)	34.48 ± 1.07	24.50 ± 1.00 **	33.06 ± 1.37	31.54 ± 0.90 ##
LV mass (mg)	88.49 ± 0.68	123.60 ± 11.45 **	78.29 ± 6.22	86.63 ± 14.26 ##

Table S3. Blood pressure in diabetic WT and IMDtg mice. SBP, DBP and MBP measured by non-invasive tail cuff system of diabetic WT and IMDtg mice. n = 6. Data are mean ± SD, ***p*<0.01 vs. CON, ##*p*<0.01 vs. HFD.

Variables	CON (n = 6)	HFD (n = 6)	IMDtg (n = 6)	IMDtg HFD (n = 6)
**SBP (mmHg)**	97.00 ± 1.16	133.50 ± 2.05 **	96.17 ± 2.5	100.00 ± 1.24 ##
**DBP (mmHg)**	68.00 ± 1.32	96.00 ± 1.93 **	67.17 ± 1.47	69.00 ± 1.65 ##
**MBP (mmHg)**	77.67 ± 1.21	108.50 ± 1.72 **	76.83 ± 0.63	79.33 ± 1.32 ##

Table S4. Glucose and lipid metabolic data of diabetic WT and IMDtg mice. Body weight, FBG, insulin levels, HOMA-IR index, triglycerides, total cholesterol, LDL-C and HDL-C in diabetic WT and IMDtg mice. n = 6-8. Data are mean ± SD, **p*<0.05, ***p*<0.01 vs. CON, #*p*<0.05, ##*p*<0.01 vs. HFD.

Variables	CON (n = 6)	HFD (n = 6)	IMDtg (n = 6-7)	IMDtg HFD (n = 6-8)
**Body weight (g)**	27.5 ± 2.35	30.67 ± 1.20 *	26.56 ± 1.47	29.41 ± 1.51
**FBG (mmol/L)**	5.78 ± 0.99	10.79 ± 1.51 **	10.01 ± 1.18	10.31 ± 0.89
**Fasting insulin (mIU/L)**	11.82 ± 0.87	16.78 ± 0.67 **	12.25 ± 0.69	12.90 ± 0.88 ##
**HOMA-IR**	3.12 ± 0.58	7.56 ± 0.99 **	5.45 ± 0.74	5.92 ± 0.73 #
**Triglycerides (mmol/L)**	1.05 ± 0.22	1.69 ± 0.29 **	1.14 ± 0.19	1.20 ± 0.15 ##
**Cholesterol (mmol/L)**	1.52 ± 0.12	4.54 ± 0.30 **	1.64 ± 0.22	2.96 ± 0.18 ##
**LDL-C (mmol/L)**	0.50 ± 0.04	1.00 ± 0.15 **	0.49 ± 0.03	0.85 ± 0.03 #
**HDL-C (mmol/L)**	1.07 ± 0.17	0.77 ± 0.15 **	1.07 ± 0.15	1.66 ± 0.18 ##

Table S5. Echocardiographic parameters of diabetic WT and IMD$^{-/-}$ mice. LVID; s, LVPW; s, LVAW; s, LV Vol; s, LVID; d, LVPW; d, LVAW; d, LV Vol; d, EF, FS and LV mass of diabetic WT and IMD$^{-/-}$ mice quantified by ultrasonography. n = 5-6. Data are mean ± SD, **p*<0.05, ***p*<0.01 vs. CON, $^{\#}$*p*<0.05, $^{\#\#}$*p*<0.01 vs. HFD.

Variables	CON (n = 5)	HFD (n = 5-6)	IMD-/- (n = 5-6)	IMD-/- HFD (n = 5)
**LVID; s (mm)**	2.95 ± 0.28	3.51 ± 0.09 *	3.10 ± 0.11	3.84 ± 0.09 ##
**LVPW; s (mm)**	0.96 ± 0.12	1.32 ± 0.06 **	0.94 ± 0.11	1.39 ± 0.10
**LVAW; s (mm)**	0.94 ± 0.08	1.25 ± 0.07 **	0.96 ± 0.12	1.40 ± 0.05 ##
**LV Vol; s (μl)**	17.75 ± 1.66	23.53 ± 1.30 *	18.27 ± 1.51	25.19 ± 1.29
**LVID; d (mm)**	3.78 ± 0.22	4.27 ± 0.07 *	3.94 ± 0.22	4.60 ± 0.23 ##
**LVPW; d (mm)**	0.90 ± 0.10	1.24 ± 0.06 **	0.91 ± 0.13	1.44 ± 0.12 #
**LVAW; d (mm)**	0.98 ± 0.06	1.23 ± 0.04 **	0.95 ± 0.07	1.38 ± 0.08 ##
**LV Vol; d (μl)**	43.16 ± 2.41	62.09 ± 1.75 **	44.26 ± 1.19	74.48 ± 4.14 #
**EF (%)**	61.09 ± 1.91	53.11 ± 2.04 *	57.92 ± 0.81	47.01 ± 0.68 #
**FS (%)**	33.15 ± 1.31	28.85 ± 0.42 *	32.70 ± 1.12	24.64 ± 0.81 ##
**LV mass (mg)**	89.49 ± 2.33	108.80 ± 7.60 *	80.38 ± 4.07	120.40 ± 2.48 #

Table S6. Blood pressure in diabetic WT and IMD$^{-/-}$ mice. SBP, DBP and MBP measured by non-invasive tail cuff system of diabetic WT and IMD$^{-/-}$ mice. n = 6. Data are mean ± SD, ***p*<0.01 vs. CON, $^{\#}$*p*<0.05, $^{\#\#}$*p*<0.01 vs. HFD.

Variables	CON (n = 6)	HFD (n = 6)	IMD-/- (n = 6)	IMD-/- HFD (n = 6)
**SBP (mmHg)**	99.33 ± 2.14	131.00 ± 2.03 **	101.50 ± 3.14	140.50 ± 1.98 ##
**DBP (mmHg)**	70.00 ± 1.32	94.33 ± 1.31 **	68.00 ± 0.93	100.00 ± 1.39 #
**MBP (mmHg)**	79.78 ± 1.34	106.60 ± 1.19 **	79.17 ± 1.53	113.50 ± 1.19 ##

Table S7. Glucose and lipid metabolic data of diabetic WT and IMD$^{-/-}$ mice. Body weight, FBG, insulin levels, HOMA-IR index, triglycerides, total cholesterol, LDL-C and HDL-C in diabetic WT and IMD$^{-/-}$ mice. n = 6-13. Data are mean ± SD, **p*<0.05, ***p*<0.01 vs. CON, $^{\#}$*p*<0.05, $^{\#\#}$*p*<0.01 vs. HFD.

Variables	CON (n = 6)	HFD (n = 6)	IMD-/- (n = 6-10)	IMD-/- HFD (n = 6-13)
**Body weight (g)**	26.83 ± 2.64	29.82 ± 1.35 *	28.07 ± 2.07	29.45 ± 2.50
**FBG (mmol/L)**	6.15 ± 0.65	9.02 ± 1.00 **	5.94 ± 0.81	13.15 ± 1.76 ##
**Fasting insulin (mIU/L)**	11.82 ± 0.87	16.78 ± 0.67 **	12.46 ± 0.58	19.04 ± 1.29 ##
**HOMA-IR**	3.12 ± 0.58	7.56 ± 0.99 **	5.01 ± 2.41	11.08 ± 1.17##
**Triglycerides (mmol/L)**	1.23 ± 0.12	1.58 ± 0.16 **	1.32 ± 0.09	1.94 ± 0.15 ##
**Cholesterol (mmol/L)**	1.66 ± 0.23	4.45 ± 0.28 **	1.46 ± 0.19	5.44 ± 0.57 ##
**LDL-C (mmol/L)**	0.53 ± 0.07	0.96 ± 0.04 **	0.47 ± 0.10	0.91 ± 0.04
**HDL-C (mmol/L)**	1.17 ± 0.19	0.88 ± 0.15 *	0.98 ± 0.10	1.06 ± 0.25

Table S8. Primer sequences for real-time PCR.

Target		Sequence	Annealing temperature (°C)
Adm2	Sense	5'-GCTGATGGTCACGGTAACCC-3'	60
**(mouse)**	Antisense	5'-TTCCAGACTACAGGCTGAAGG-3'	
Adm2	Sense	5'-GTCAAGTCCAGAATCTCAGCCATCG-3'	60
**(rat)**	Antisense	5'-ACAGCACTCTAGGCGGATACC-3'	
Calcrl	Sense	5'-CCTGGGACGGATGGCTATG-3'	60
**(mouse)**	Antisense	5'-ACTTTCTCATGCGTGCTGTTAT-3'	
Calcrl	Sense	5'-AGAGCCTAAGTTGCCAACGGA-3'	60
**(rat)**	Antisense	5'-CTTCTCCGCAAACACAGCCA-3'	
Ramp1	Sense	5'-GAGACTATTGGGAAGACGCTATG-3'	60
**(mouse)**	Antisense	5'-CTCCTCCAGACCACCAGTG-3'	
Ramp1	Sense	5'-CACCACCGCTACTTCAGCAA-3'	60
**(rat)**	Antisense	5'-GGCAGTCATGAGCAGTGTGAC-3'	
Ramp2	Sense	5'-GCAGGCATTACAGCGACCT-3'	60
**(mouse)**	Antisense	5'-GGAGCAGTTCGCAAAGTGTATC-3'	
Ramp2	Sense	5'-TGCTTGGAGTACGAGGCAGA-3'	60
**(rat)**	Antisense	5'-GAAGGTAGGCTGCACCAAGG-3'	
Ramp3	Sense	5'-GTGAGTGTGCCCAGGTATGC-3'	60
**(mouse)**	Antisense	5'-AGGTTGCACCACTTCCAGAC-3'	
Ramp3	Sense	5'-GGCAAGGTCATCTGGAAGGTGTG-3'	60
**(rat)**	Antisense	5'-TAGCCACGGTCAACAAGACTG-3'	
Nppa	Sense	5'-TTGGAGCCCAGAGTGGACTA-3'	60
**(mouse)**	Antisense	5'-ACACACCACAAGGGCTTAGG-3'	
Nppa	Sense	5'-ATCTGCCCTCTTGAAAAGCA-3'	60
**(rat)**	Antisense	5'-GGATCTTTTGCGATCTGCTC-3'	
Nppb	Sense	5'-ATCTCCTGAAGGTGCTGTCC-3'	60
**(mouse)**	Antisense	5'-TGCATCTTGAATTGCTCTGG-3'	
Nppb	Sense	5'-GACGGGCTGAGGTTGTTTTA-3'	60
**(rat)**	Antisense	5'-ACTGTGGCAAGTTTGTGCTG-3'	

Target		Sequence	Annealing temperature (°C)
Col1a1	Sense	5’-ATCCTGCCGATGTCGCTAT-3’	60
**(mouse)**	Antisense	5’-CCACAAGCGTGCTGTAGGT-3’	
Col1a1	Sense	5’-TGAACGTGACCAAAAACCAA-3’	60
**(rat)**	Antisense	5’-AAGGAACAGAAAAGGCAGCA-3’	
Col3a1	Sense	5’-CATGACTGTCCCACGTAAGCA-3’	60
**(mouse)**	Antisense	5’-ATTCGCCTTCATTTGATCCCA-3’	
Col3a1	Sense	5’-GTCCACGAGGTGACAAAGGT -3’	60
**(rat)**	Antisense	5’-CATCTTTTCCAGGAGGTCCA-3’	
Cpt1b	Sense	5’-CATGTATCGCCGCAAACTGG-3’	60
**(mouse)**	Antisense	5’-CCTGGGATGCGTGTAGTGTT-3’	
Cpt1b	Sense	5’-GCGGAAGCACACCAGGCAGTA-3’	60
**(rat)**	Antisense	5’-ATGTTTGGAAGCTATAGAGCA-3’	
Cpt1a	Sense	5’-GGTCTGGCTCTACCACGATG-3’	60
**(rat)**	Antisense	5’-CTCCGATTCGTCCAACGTCA-3’	
Gapdh	Sense	5’-AAGAAGGTGGTGAAGCAG-3’	60
**(mouse)**	Antisense	5’-TCATACCAGGAAATGAGC-3’	
Gapdh	Sense	5’-AGACAGCCGCATCTTCTTGT-3’	60
**(rat)**	Antisense	5’-CTTGCCGTGGGTAGAGTCAT-3’	
